# Single-cell epigenomic reconstruction of developmental trajectories from pluripotency in human neural organoid systems

**DOI:** 10.1038/s41593-024-01652-0

**Published:** 2024-06-24

**Authors:** Fides Zenk, Jonas Simon Fleck, Sophie Martina Johanna Jansen, Bijan Kashanian, Benedikt Eisinger, Małgorzata Santel, Jean-Samuel Dupré, J. Gray Camp, Barbara Treutlein

**Affiliations:** 1 Department of Biosystems Science and Engineering, ETH Zürich, Basel, Switzerland; 2 Institute of Human Biology (IHB), Roche Pharma Research and Early Development, Roche Innovation Center Basel, Basel, Switzerland; 3 Present Address: Brain Mind Institute, School of Life Sciences EPFL, Lausanne, Switzerland

**Keywords:** Cell fate and cell lineage, Epigenomics, Developmental neurogenesis, Epigenetics

## Abstract

Cell fate progression of pluripotent progenitors is strictly regulated, resulting in high human cell diversity. Epigenetic modifications also orchestrate cell fate restriction. Unveiling the epigenetic mechanisms underlying human cell diversity has been difficult. In this study, we use human brain and retina organoid models and present single-cell profiling of H3K27ac, H3K27me3 and H3K4me3 histone modifications from progenitor to differentiated neural fates to reconstruct the epigenomic trajectories regulating cell identity acquisition. We capture transitions from pluripotency through neuroepithelium to retinal and brain region and cell type specification. Switching of repressive and activating epigenetic modifications can precede and predict cell fate decisions at each stage, providing a temporal census of gene regulatory elements and transcription factors. Removing H3K27me3 at the neuroectoderm stage disrupts fate restriction, resulting in aberrant cell identity acquisition. Our single-cell epigenome-wide map of human neural organoid development serves as a blueprint to explore human cell fate determination.

## Abstract (web summary)

The mechanisms underlying human cell diversity are unclear. Here the authors provide a single-cell epigenome map of human neural organoid development and dissect how epigenetic changes control cell fate specification from pluripotency to distinct cerebral and retina neural types.

## Main

During development and differentiation, cells undergo remarkable cell fate transitions, starting from pluripotent cells and multipotent progenitors to give rise to all major cell types of the body. During these transitions, developmental plasticity becomes increasingly restricted until the cells stop dividing and acquire their terminal fate. Despite recent progress in the field of epigenetics and reprogramming, the details of how human cells acquire and maintain their identity remain elusive. What is known is that, during differentiation, histone modifications are involved in the packaging of DNA on nucleosomes and can either directly or indirectly influence the expression of underlying genes^1^. In vivo, chromatin regulation during differentiation is dynamic and complex, and recent studies have shown that mutations within histone tails or enzymes that modify the histone tails are involved in neurodevelopmental disorders, which emphasize their importance in the developing nervous system^2,3^. Previous bulk studies of histone modifications in human developing tissues failed to capture the individual trajectories of furcating fates^4^. In the present study, we explore how epigenetic changes on chromatin are involved in cell fate decisions during the human brain and retina development using organoids and a primary developing human brain as validation. We include H3K27me3 as a repressive mark at developmental genes^5^, H3K27ac as a mark of active enhancers and promoters^6,7^ and H3K4me3 as a mark of active or bivalent promoters of developmental genes^5,8,9^. In addition, these marks interact with one another and can act in concert or be mutually exclusive^10,11^. We provide an atlas of these marks over organoid development and identify different modes of epigenetic regulation during fate restriction and neuronal specification. We integrate all modalities and explore dynamics over differentiation trajectories from pluripotency using gene regulatory network (GRN) analysis and find that perturbation of a non-redundant member of the PRC2 complex, embryonic ectoderm development (EED), which disrupts the writing of H3K27me3 in the early neuroepithelium, results in loss of fate restriction and the emergence of aberrant cell states.

## Results

### Single-cell epigenomic atlas of brain organoid development

**Fig. 1:**
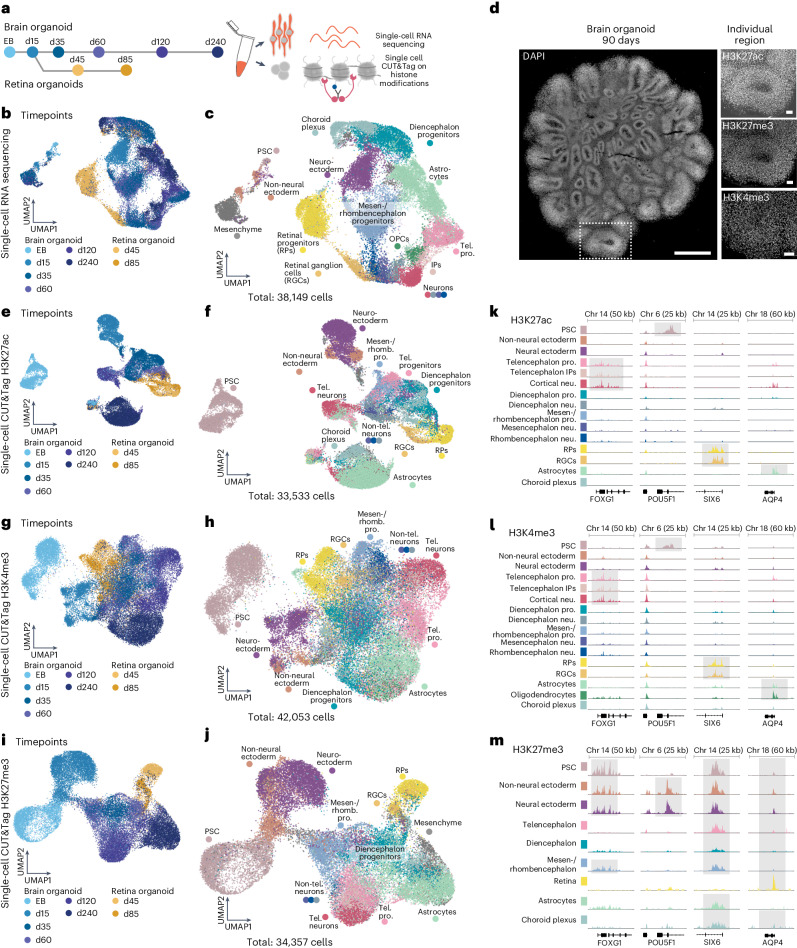
Single-cell epigenomic atlas of human brain organoid development from pluripotency to neurogenesis. **a**, Experimental outline. scCUT&Tag and scRNA-seq were performed at different developmental timepoints during brain and retina organoid development (number of pooled organoids per timepoint: brain: EB (d5): 500, d15: 150, d35: 50, d60: 25, d120: 20, d240: 20, retina: d45: 50, d85: 50). **b**, Dimensionality reduction and embedding with UMAP of scRNA-seq data with cells colored based on developmental timepoint reveals heterogeneity of cell states and the neuroepithelium as a branching point. **c**, UMAP embedding of scRNA-seq data with cells colored and labeled by cell state (IP, intermediate progenitor; RGC, retinal ganglion cell; RP, retinal progenitor). **d**, DAPI staining of an organoid at day 90; scale bar, 1,000 µm (left). One ventricle of an organoid stained for H3K27ac, H3K27me3 and H3K4me3; scale bar, 100 µm (right) (Methods). This is a representative image. The experiment was performed three times on biological replicates. **e**–**j**, UMAP embedding of scCUT&Tag data for H3K27ac (**e**,**f**), H3K4me3 (**g**,**h**) and H3K27me3 (**i**,**j**) colored and labeled by timepoint (**e**,**g**,**i**) or cell state (**f**,**h**,**j**). **k**–**m**, Genome browser snapshots of the enrichment of the respective mark (H3K27ac (**k**) and H3K4me3 (**l**)—enriched at active genes; H3K27me3—enriched at repressed genes (**m**)) at four different marker genes (FOXG1—telencephalon, POU5F1—pluripotency, SIX6—retina and AQP4—astrocytes). Each signal track represents the summarized signal of all cells of the annotated cell state. Shaded areas highlight the detected peaks. d, day; Tel., telencephalon; rhomb., rhombencephalon; pro., progenitors; neu., neurons.

**Extended Data Fig. 1:**
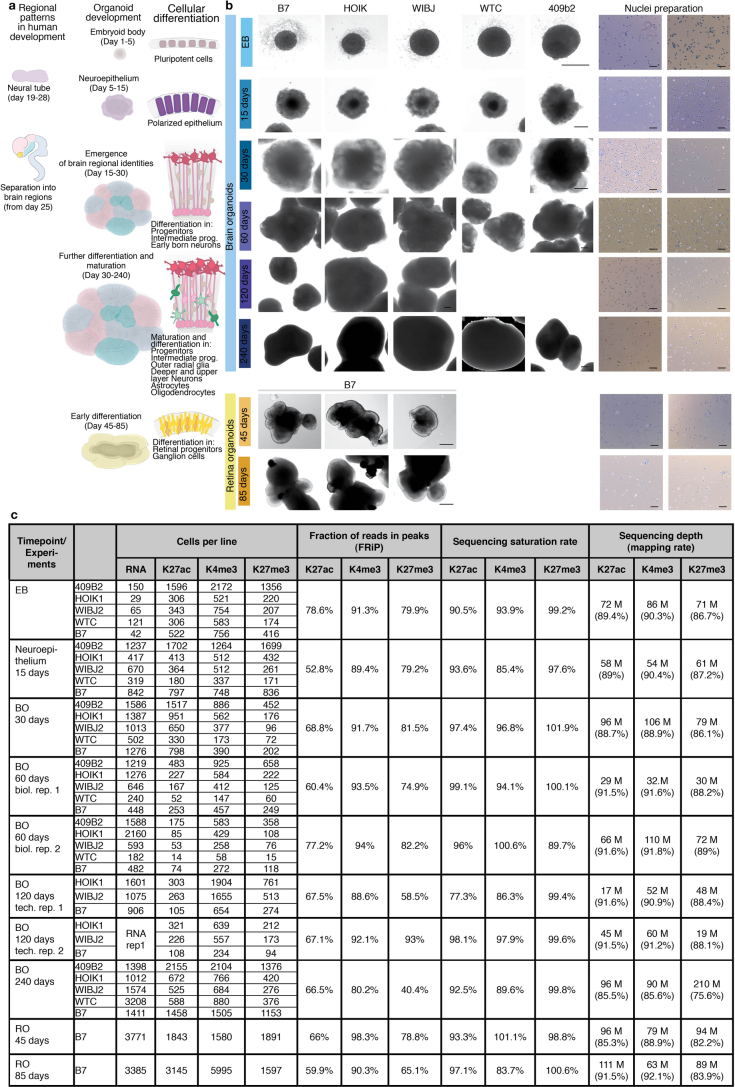
Epigenetic profiling of brain organoid development. **a**, Schematic of brain and retina organoid development in relation to early human brain development and emergence of cell state heterogeneity. **b**, Brightfield images of organoids during development (scale bar 500 µm) and the corresponding representative images of nuclei suspensions without adjusting the concentration (scale bar 100 µm). These are representative images of multiple experiments, each preparation has been performed multiple times and each organoid batch contained up to 96 organoids per cell line. **c**, Table giving an overview of the samples, cell lines and time points processed in the presented analysis including important quality control metrics like recovered number of cells per cell line, the fraction of reads in peaks, the sequencing saturation rate as well as total number of reads per sample and fraction of reads that maps to the reference genome (BO-Brain Organoids, RO-Retina Organoids, K27ac – H3K27ac, K4me3 – H3K4me3, K27me3 – H3K27me3).

To dissect the role and dynamic turnover of histone modifications during human brain and retinal organoid development, we performed CUT&Tag and mRNA sequencing in single cells (scCUT&Tag and scRNA-seq) on a timecourse, covering cell fate transitions from induced pluripotent stem cells (iPSCs) to terminally differentiated neurons and glial cells. We recorded three histone modifications (H3K27ac, H3K27me3 and H3K4me3) and RNA expression from the same single-cell suspensions of five different cell lines at six developmental timepoints in brain organoids (days 5, 15, 35, 60, 120 and 240) and of one cell line at two timepoints in retina organoids (days 45 and 85) (Fig. 1a and Extended Data Fig. 1a–c). Retinal cells emerge from the developing diencephalic neuroepithelium and can spontaneously arise in unguided brain organoids, supporting the inclusion of both organoid systems in a neural epigenomic trajectory reconstruction.

Dimensionality reduction and embedding with uniform manifold approximation and projection (UMAP)^12^ using the scRNA-seq data (38,149 cells) revealed diverse populations at each timepoint. We annotated clusters by comparing gene expression to reference datasets^13,14^ and analyzing marker gene expression. The cells represented in the dataset cover transitions from early pluripotent stages at day 5 to a stratified neuroepithelium at day 15, with progenitors diversifying into retina and brain regional identities (telencephalon, diencephalon and non-telencephalon) between days 35 and 60 (Fig. 1b,c). Brain region-specific and retinal neurons start to develop from day 35 and increase in abundance over time (Fig. 1b,c), and, by day 120, astrocytes and oligodendrocyte precursor cells (OPCs) appear, coinciding with the gliogenic switch during the second trimester of human embryonic development (Fig. 1b,c)^15,16^.

We investigated the spatial distribution of H3K27ac, H3K27me3 and H3K4me3 by immunofluorescence and found that all nuclei stain for the three histone modifications (Fig. 1d). We established scCUT&Tag in brain organoids and recorded the genome-wide distribution of H3K27ac (33,533 cells), H3K27me3 (34,357 cells) and H3K4me3 (42,053 cells) in the same cell suspensions as used for the scRNA-seq experiments. We obtained high-quality data from all experiments as reflected by the fraction of reads in peaks (Extended Data Fig. 1c), the nucleosome pattern and the comparably high number of fragments (median: H3K4me3, 722; H3K27ac, 641; H3K27me3, 302) recovered from each cell (Supplementary Fig. 1a–d)^17–19^. Dimensionality reduction and embedding with UMAP revealed remarkable cell state diversity over the timecourse for each modality (Fig. 1e–j).

**Extended Data Fig. 2:**
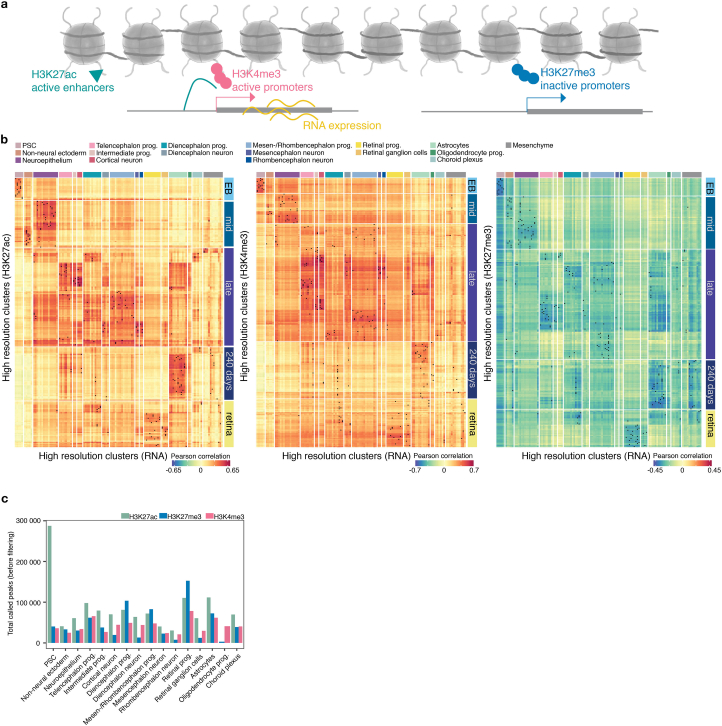
Multi-modal matching between high-resolution clusters and cell type annotation. **a**, Schematic of the profiled modalities and their functional molecular role. H3K27ac marks active promoters and enhancers, H3K4me3 marks active promoters. H3K27me3 marks repressed regions with inactive promoters. **b**, Heatmaps showing correlation of high-resolution clusters across modalities. Clusters matched by bipartite matching during integration are indicated with a cross (see Methods ‘Matching of scRNA and scCUT&Tag’ for details). **c**, Barplot showing the total number of detected peaks for each histone modification in each cell state (H3K27ac: PSC-287442, NNE-40770, NE-60818, Tel NPC-97930, Tel IP-79388, Cort Neu-0137, Dien NPC- 81152, Dien Neu-3836, MRh NPC-72112, Mesen Neu-40315, Rhom Neu-30485, RPC-110547, RGC-60662, Ast-111708, ChP-69642, H3K27me3: PSC-40267, NNE-33245, NE-30462, Tel NPC-61953, Tel IP-37903, Cort Neu-19555, Dien NPC-103617, Dien Neu-13200, MRh NPC-82809, Mesen Neu-22834, Rhom Neu-7681, RPC-152647, RGC-12307, Ast-72446, OPC-2934, ChP-38992, H3K4me3: PSC-35922, NNE-25000, NE-34006, Tel NPC-65608, Tel IP-26895, Cort Neu-44418, Dien NPC-49196, Dien Neu-43978, MRh NPC-47889, Mesen Neu-24198, Rhom Neu-21049, RPC-78329, RGC-29680, Ast-61828, OPC-40994, ChP-40390).

**Extended Data Fig. 3:**
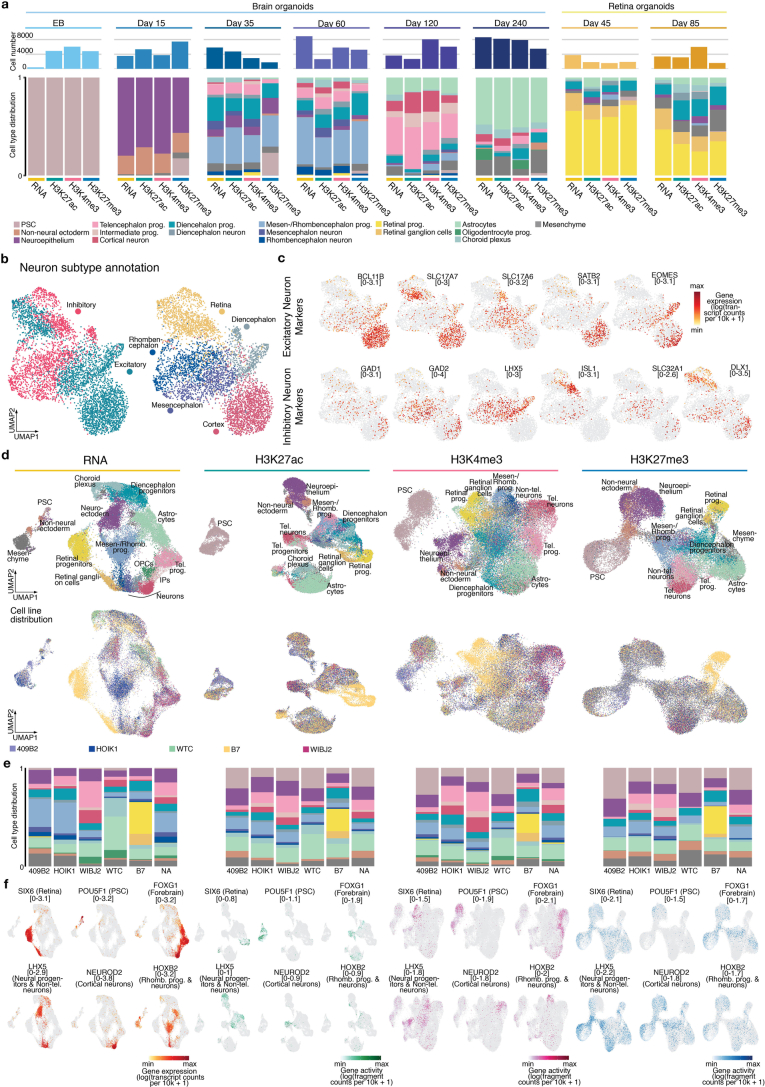
Cell type distribution across modalities and cell lines. **a**, Barplot of absolute cell number per time point (top panel) for all modalities. Stacked barplot of cell type fraction within each time point (bottom panel) for all modalities. **b**, UMAP embedding of neuronal populations in the time course colored by excitatory and inhibitory identity (left panel) or brain region of origin (right panel). **c**, UMAP embedding as in b colored by the expression (log(transcript counts per 10k + 1) of excitatory neuronal subtype markers (top panel) and inhibitory neuronal subtype markers (bottom panel). **d**, UMAP embedding of all modalities in the developmental time course colored by cell state (top panel) and cell line (bottom panel). **e**, Stacked barplot showing cell type fractions for each cell line in the dataset. This shows that all cell lines contribute to all major regional branches and cell states. Retina organoids have only been derived from the B7 cell line. **f**, Same UMAP as in d colored by expression (log(transcript counts per 10k + 1); in case of RNA-seq) and gene activity (log(fragment counts per 10k + 1 on gene body +2 kb promoter region); in case of CUT&Tag) of genes marking different regional identities and cell states (Non-tel. – Non-telencephalon).

To annotate cell states within the epigenomic datasets, we first performed a high-resolution Louvain clustering^20^ for each modality separately to enhance robustness through increasing fragment counts per modality (H3K27me3, 104,000 mean fragments; H3K27ac, 236,000 mean fragments; H3K4me3, 417,000 mean fragments). Next, we compared each epigenomic high-resolution cluster with the annotated scRNA-seq clusters using minimum-cost, maximum-flow (MCMF) bipartite matching^21^ based on correlation for activating histone marks (H3K27ac and H3K4me3) and anti-correlation for the repressive mark H3K27me3 (Extended Data Fig. 2a,b). Based on these matches, we transferred annotated cell labels from RNA to the other modalities, obtaining a high number of peaks per cell state (Extended Data Fig. 2c). After label transfer, cell state proportions were similar among all modalities, and even rare populations, such as intermediate progenitors, could be recovered in the histone modification dataset, supporting integration accuracy (Extended Data Fig. 3a). Next, we examined neuronal cell state heterogeneity and found inhibitory and excitatory neurons in all regional branches (Extended Data Fig. 3b,c) with the exception of the telencephalon branch, which contained a strong majority of excitatory neurons. The different cell lines showed similar cell type distributions, and all lines contributed to all different cell states and brain regional identities, indicating reproducibility of the differentiation across cell lines (Extended Data Fig. 3d,e). We detected highly enriched signal (peaks) of activating marks at known regulators of cell type and regional identity (for example, FOXG1, telencephalic branch; NEUROD2, telencephalic neurons; SIX6, retinal branch; LHX5, neuroepithelium and non-telencephalon neurons; HOXB2, rhombencephalic branch; POU5F1, pluripotent stem cells (PSCs); and AQP4, astrocytes) (Fig. 1k,l and Extended Data Fig. 3f) and found H3K27me3 signal (repressive) in cell states that lack expression of the respective genes (Fig. 1m). To our knowledge, these data provide the first comprehensive epigenomic atlas of human central nervous system development in organoids containing activating and repressive histone modifications along with RNA expression on the single-cell level. This resource can also be browsed interactively at https://episcape.ethz.ch.

### Epigenomic switches during regional diversification

**Fig. 2:**
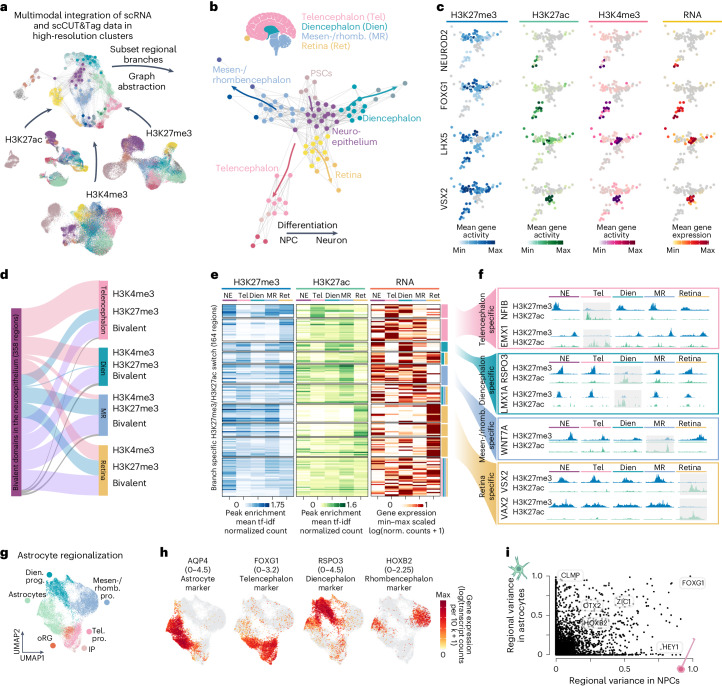
Integration of chromatin and RNA modalities reveals epigenetic switches during brain region diversification. **a**, Schematic of the multimodal data integration through high-resolution cluster matching between histone marks and RNA (see Extended Data Fig. 2b and the ‘Matching of scRNA and scCUT&Tag’ subsection for details). **b**, Schematic of the human brain (top) and force-directed layout (bottom) of the matched high-resolution clusters colored by regional branch and cell state; it shows the neuroepithelium as a branching point for regional identities. **c**, Force-directed layout colored by RNA expression and gene activities (abundance of histone modifications at the gene and an extended promoter region +2 kb upstream) of all three chromatin marks for NEUROD2, FOXG1 (telencephalon), LHX5 (non-telencephalon) and VSX2 (retina). **d**, Alluvial plot of bivalent H3K27me3 and H3K4me3 peaks at the neuroepithelium stage. Most peaks get resolved and become enriched for either of the two marks in a specific brain-regional branch (blue-H3K27me3 and pink-H3K4me3). Peaks that stay bivalent in any of the brain regions are labeled in purple. **e**, Heatmap of signal enrichment of region-specific peaks showing switching between H3K27me3 and H3K27ac. Expression of the closest gene is shown in the right panel (Dien, diencephalon; MR, mesen-/rhombencephalon; NE, neuroepithelium; Ret, retina; Tel, telencephalon). **f**, Genomic tracks showing switching peaks close to region-specific genes (blue-H3K27me3 and green-H3K27ac). **g**, UMAP embedding of NPCs and astrocytes from the 1-month to the 8-month timepoints within the dataset (IP, intermediate progenitor; oRG, outer radial glia). **h**, UMAP embedding colored by gene expression of a general astrocyte marker (AQP4) and regional marker genes (FOXG1, telencephalon; RSPO3, diencephalon; HOXB2, rhombencephalon). **i**, Scatter plot showing the regional variance of gene expression for NPCs and astrocytes (see Methods, ‘Analysis of pseudotemporal and regional variance’ subsection, for details).

**Extended Data Fig. 4:**
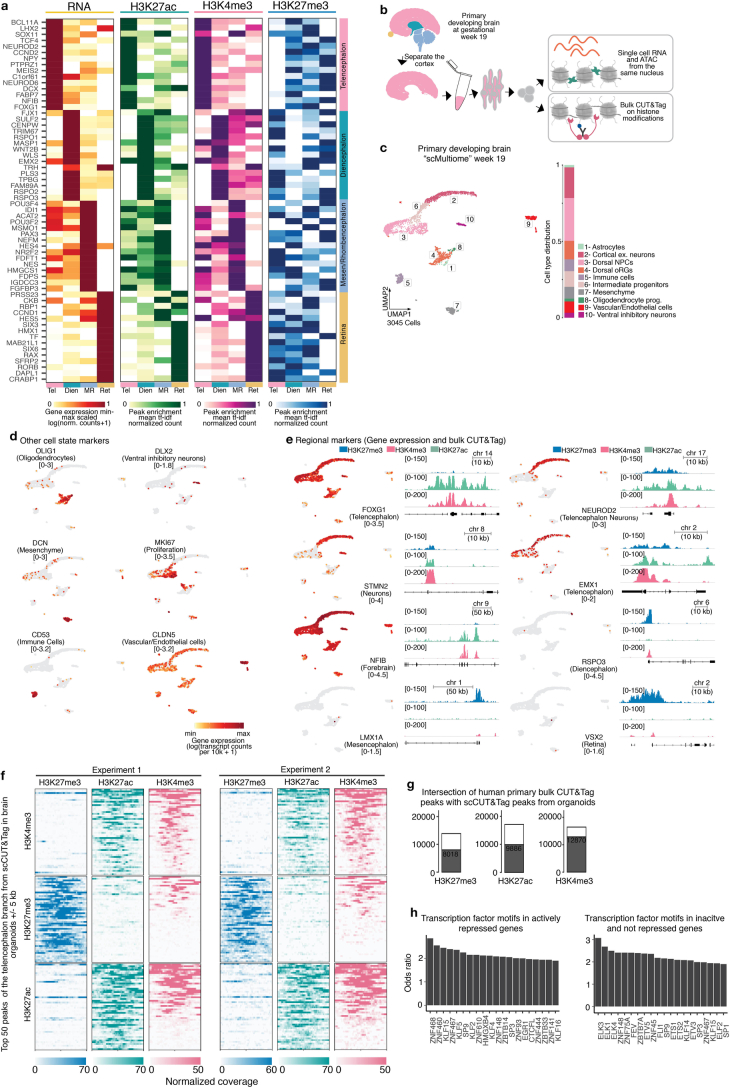
Single-cell ‘multiome’ and bulk CUT&Tag data of human developing cortex at gestation week 19 validating the regulation of regional identity markers in the telencephalon lineage. **a**, Heatmap showing gene expression (log(transcript counts per 10k + 1) and gene activity (log(fragment counts per 10k + 1 on gene body +2 kb promoter region) of the top 15 regional markers in the different brain regional branches of the time course. **b**, Schematic of the experimental setup. **c**, UMAP embedding of the single-cell multiome (co-measurement of scRNA-expression and sc-chromatin accessibility) data from the human developing brain colored by annotated cell states (right). Stacked barplot, showing the cell state distribution within the sample, reveals that most cells originate from the telencephalon. **d**, UMAP embedding as in (c) colored by gene expression of cell state markers used to annotate the different cell populations. **e**, Regional markers identified in Fig. 2e,f recapitulate the switching of activating and repressing marks in signal tracks of bulk H3K27me3, H3K27ac and H3K4me3 CUT&Tag data from a primary developing telencephalon (FOXG1, NEUROD2, STMN2, EMX1, NFIB) while non-telencephalon regional markers remain repressed (RSPO3, LMX1A, VSX2). **f**, Heatmaps showing the bulk CUT&Tag enrichment from the primary developing human brain on the highest enriched telencephalon peaks (+/−5 kb) from the scCUT&Tag organoid time course per modality. This shows that the chromatin state between regional marker genes in the telencephalon branch is similar between organoid and primary tissue. **g**, Intersection of human primary bulk CUT&Tag peaks with scCUT&Tag peaks from organoids, showing overlap between the organoids and primary tissue. **h**, Transcription factor motif enrichment in the gene sets that are actively repressed by H3K27me3 (left panel) and inactive but not repressed (right panel), showing potential regulators of inactive chromatin in the early developing brain.

To visualize the branching of neuroepithelial cells into neuronal progenitor cells (NPCs) and neurons of different brain regions, we computed terminal fate probabilities based on RNA expression for each regional identity using CellRank^22,23^. We summarized these probabilities for each high-resolution cluster and used them to construct a graph representation of the differentiation events (Fig. 2a,b). A force-directed layout of this graph revealed the transition of PSCs into neuroepithelium, which then diversified into branches of region-specific neurogenesis (Dien, diencephalon; MRh, mesen-/rhombencephalon; Ret, retina; Tel, telencephalon) (Fig. 2b). By mapping the matched high-resolution clusters of chromatin modalities (Fig. 2a and Extended Data Fig. 2b) onto this representation, we could visualize histone modifications switching between neuroepithelium and brain region branches (Fig. 2c). For each chromatin modification, we computed a gene activity score by summarizing fragment counts over the gene body plus an extended promoter region. We first selected the top 15 regulators that showed differential enrichment between regions (Extended Data Fig. 4a). Visualizing gene activities on the graph layout revealed that region-specific transcription factors (for example, FOXG1, NEUROD2, LHX5 and VSX2) tend to be broadly repressed by H3K27me3 outside of their expression domain and show low levels of H3K4me3 (Fig. 2c). To validate the epigenetic regulatory patterns for the forebrain branch, we generated single-cell multiome (scRNA-expression and scATAC-chromatin accessibility from the same single cell) and bulk CUT&Tag data from a primary developing forebrain at 19 gestational weeks (gw) (Extended Data Fig. 4b–f). We found the same enrichment of activating and repressing histone modifications on these regional regulators (Extended Data Fig. 4e). For all three histone modifications, the 50 highest enriched regions (peaks) in the telencephalon branch of the organoids showed similar enrichment in the primary developing brain sample (Extended Data Fig. 4f). Intersection of the bulk CUT&Tag peaks from the primary developing brain with the scCUT&Tag peaks from the organoids revealed a high overlap (Extended Data Fig. 4g).

Overall, we found that most protein-coding genes were either expressed or repressed at some point in the developmental timecourse (61%); some were never repressed and always active (16%); some were always inactive and repressed (15%); and some were not repressed and inactive (8%). Inactive genes were enriched for Gene Ontology (GO) terms for sensory perception, immune system, skin development and fertilization, indicating repression of non-neural programs. Interestingly, inactive genes without repressive histone modifications tended to be enriched for motifs of transcription factors that were either not expressed (FEV, FLI1 and KLF14) in the timecourse or restricted to specific cell states (ETS1). This suggests that chromatin repression is not required in instances where the corresponding transcription activator is absent or when the transcription factors act as repressors themselves (Extended Data Fig. 4h)^24,25^.

**Extended Data Fig. 5:**
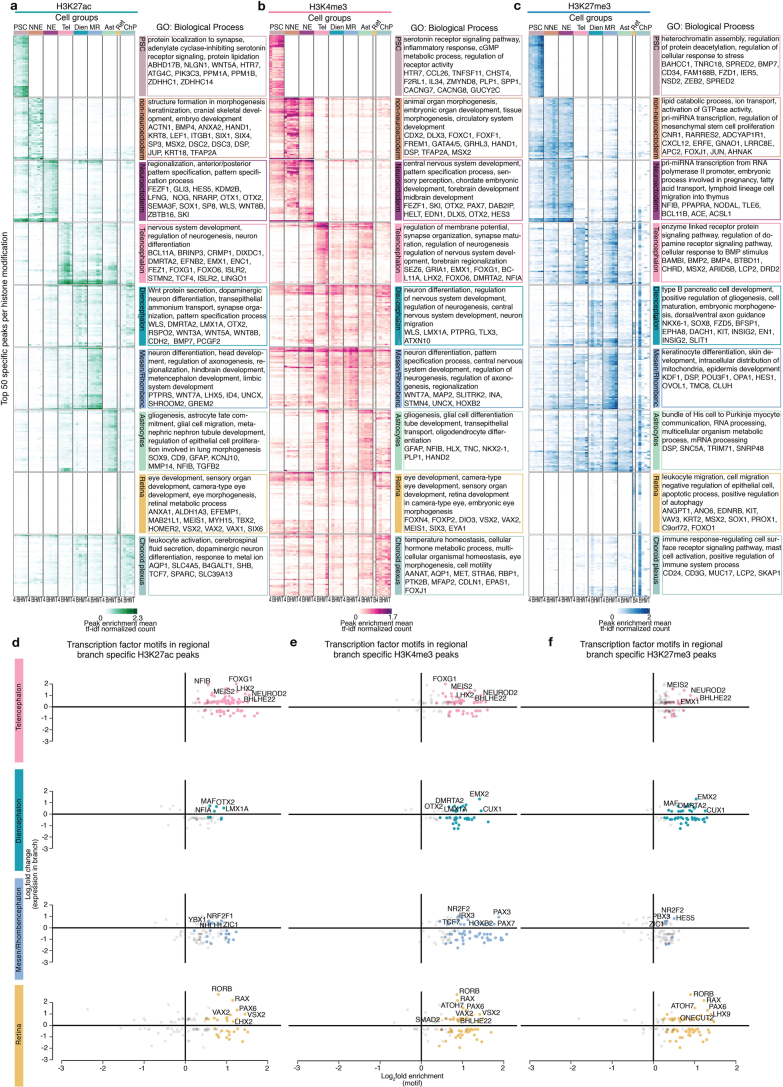
Cell type- and region-specific chromatin modification peaks and corresponding transcription factor motifs. **a**–**c**, Heatmaps showing normalized signal enrichment (mean tf-idf-normalized fragment counts) at state- and region-specific peaks for H3K27ac (**a**), H3K4me3 (**b**), and H3K27me3 (**c**) for all different cell lines used in the dataset (in order: 4- 409B2, B-B7, H-HOIK1, W-WIBJ2, T-WTC, the retina branch has been derived from B7 only) (Tf-idf, term frequency-inverse document frequency). Selected genes in proximity to these peaks and representative GO Biological Process terms are indicated on the right (see Methods ‘Functional enrichment of regulatory regions’ for details). **d**–**f**, Scatter plots showing log2 fold enrichment of transcription factor motifs in region-specific peaks H3K27ac (**d**), H3K4me3 (**e**), and H3K27me3 (**f**) versus their expression fold change in the respective regions. Colored dots indicate a motif enrichment Fisher exact test FDR < 0.01.

We investigated chromatin modifications during the formation of different brain regions from the neuroepithelium, identifying differential histone modifications across regions and cell types. We found consistent patterns between different cell lines, suggesting similar epigenetic programs during branch specification (Extended Data Fig. 5a–c and Supplementary Table 1). Ontology analysis^26^ of genes in close proximity to H3K4me3 and H3K27ac peaks revealed a strong enrichment in developmental processes. In particular, activating H3K4me3 and H3K27ac peaks had enrichments for receptor signaling and metabolic processes (PSCs), embryonic development and morphogenesis (non-neural ectoderm (NNE)) and patterning and nervous system development (neuroepithelium). Within the branches of regional neurogenesis and astrocyte differentiation, activating peaks were found enriched near genes associated with neuron maturation and differentiation (Tel), brain regional development (MRh), eye and sensory organ development (Ret), gliogenesis and glia cell differentiation (astrocytes (Ast)), cerebrospinal fluid production as well as metabolic and immune processes (choroid plexus (Chp)) (Extended Data Fig. 5a,b). In contrast to H3K4me3 and H3K27ac modifications, repressive H3K27me3 domains were found at developmental regulators not involved in brain development. We found H3K27me3 peaks close to genes associated with heterochromatin assembly or regulation of cellular stress (PSC), mesenchymal stem cell proliferation (NNE), BMP signaling (Tel) and regulation of other cell fates, such as pancreatic cells or keratinocytes (Dien and MRh) (Extended Data Fig. 5c and Supplementary Table 2). We analyzed transcription factor motifs enriched in the branch-specific peaks for each histone modification (Extended Data Fig. 5d–f and Supplementary Table 3). We found that motifs were enriched for regional regulators, such as FOXG1, NEUROD2 (Tel), OTX2, EMX2, LMX1A (Dien), HOXB2, ZIC1, PAX7 (MRh) and PAX6, VSX2 and VAXs (Ret).

**Extended Data Fig. 6:**
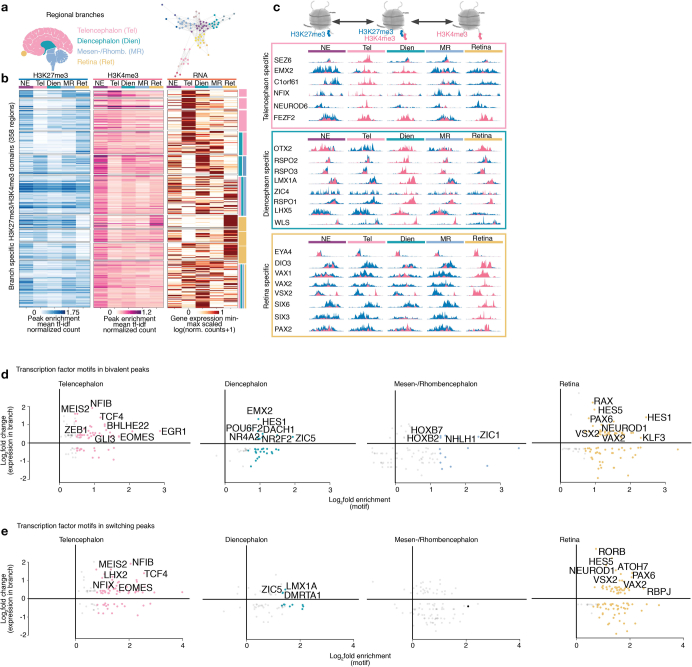
Cell type- and region-specific chromatin modification peaks. **a**, Schematic of the human brain colored by brain region (left) and force directed-layout of the main regional branches, colored by annotated brain region and developmental stage (right). **b**, Heatmap of peak enrichment (tf-idf-normalized fragment counts) of lineage-specific peaks showing co-occurrence of H3K27me3 and H3K4me3 marks in the neuroepithelium and switch to activator (H3K4me3) or repressor (H3K4me3) in a regional branch. Expression of the closest gene is shown in the right panel (min-max scaled log(transcript counts per 10k + 1)). **c**, Overlaid genomic tracks showing enrichment of bivalent peaks close to lineage-specific genes in the neuroepithelium that switch to activation or repression in the regional branches (overlaid blue-H3K27me3, magenta-H3K4me3). **d**, Scatter plot showing transcription factor motif enrichment in the H3K27me3 and H3K4me3 co-marked peaks plotted against their expression fold change in the respective regions (see Methods ‘Transcription factor motif enrichment’). Colored dots indicate a motif enrichment Fisher exact test FDR < 0.01. **e**, Scatter plot showing transcription factor motif enrichment in the H3K27me3 and H3K27ac switching peaks plotted against their expression fold change in the respective regions. Colored dots indicate a motif enrichment Fisher exact test FDR < 0.01.

Chromatin domains defined by H3K27me3 and H3K4me3 co-enrichment at repressed genes have been termed bivalent and were previously reported to occur at genes that require rapid activation during development^8,9,27^. We observed abundant H3K27me3 and H3K4me3 co-marked chromatin domains within the matched high-resolution clusters at the neuroepithelium stage (Fig. 2c,d, Extended Data Fig. 6a–c and Supplementary Table 4). We refer to these domains as bivalent and found that approximately 90% of them show full sequence overlap between both histone modifications. Several transcription factor binding motifs were enriched (Extended Data Fig. 6d and Supplementary Table 5), among them known regional regulators as well as EGR1, an immediate-early gene previously described to be involved in epigenetic remodeling^28,29^. Bivalent domains became activated (installation of H3K4me3 and removal of H3K27me3) predominantly in the telencephalon and diencephalon branch, whereas most bivalent peaks acquired repression (H3K27me3) in the mesen-/rhombencephalon (Fig. 2d). A smaller subset of domains remained bivalent as cells transitioned into the regional branches. We next analyzed switching between H3K27me3 and H3K27ac, which are considered mutually exclusive as they are installed on the same histone H3 lysine 27 residue and rarely co-occur within a nucleosome^5,6,11^. Indeed, we did not find co-enrichment of H3K27ac and H3K27me3, suggesting that the high-resolution clusters can discriminate between switching and co-enrichment. We identified a set of repressed H3K27me3 peaks during the neuroepithelial stage that switched into an active state (depletion of H3K27me3 and enrichment of H3K27ac) in individual regional branches (Fig. 2e and Supplementary Table 6). This set of peaks showed enrichment for many known regional regulators and overlapped well with the motifs called from the H3K4me3/H3K27me3 co-regulated peaks, indicating lineage-specific activation (Extended Data Fig. 6e and Supplementary Table 7). This epigenetic activation in individual regional branches was accompanied by region-specific expression of nearby genes (Fig. 2e), including important regulators of regional identity, such as EMX1 and NFIB (Tel), RSPO3 and LMX1A (Dien), WNT7A (MRh) and VSX2 and VAX2 (Ret) (Fig. 2f). We found that 60% of these genes showed previous bivalency within the neuroepithelium.

Next, we wanted to analyze regionalization also within the glial lineage. We integrated all NPCs and astrocytes from the data of 1-month-old to 8-month-old organoids. This revealed a small population of outer radial glia cells within the forebrain branch (Fig. 2g and Supplementary Fig. 2a–f). In addition, we observed region-specific gene expression in astrocytes within the organoids (Fig. 2g,h). We calculated the regional variance of gene expression between NPCs and astrocytes from different regions (Fig. 2i) and found that FOXG1, OTX2, HOXB2 and LHX2 as well as the ZIC and IRX family define regionalization in both neuron and astrocyte populations, similar to observations in mouse and primary human tissue^30–33^ (Supplementary Fig. 2a–j).

### Characterizing regulatory elements in the developmental timecourse

**Fig. 3:**
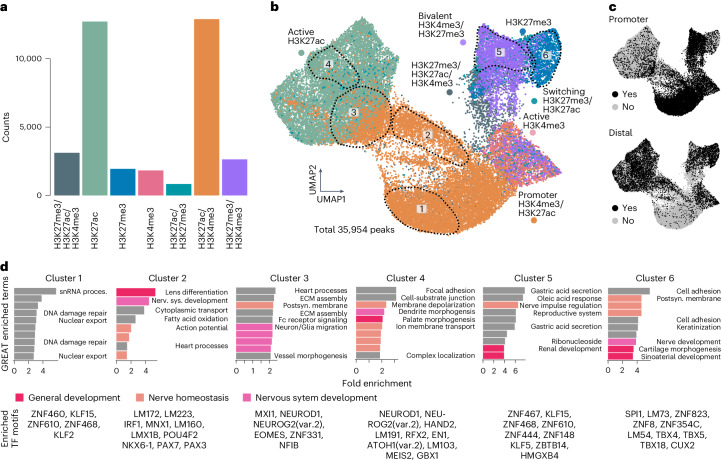
Landscape of regulatory elements during neural organoid development. **a**, Bar plot showing counts of different peak classes within the dataset, filtered by detection in more than 10% of the cells for each group and more than 50 high-resolution clusters (H3K27me3, H3K27ac and H3K4me3: 3,116; H3K27ac: 12,714; H3K27me3: 1,941; H3K4me3: 1,821; H3K27ac and H3K27me3: 831; H3K27ac and H3K4me3: 12,894; H3K27me3 and H3K4me3: 2,637). **b**, UMAP embedding of regulatory regions identified in the developmental timecourse based on their detection rates in high-resolution clusters colored by peak class. The encircled clusters were identified by Louvain clustering (Extended Data Fig. 7f); GREAT enrichment and transcription factor motif enrichment in selected and encircled clusters are shown in **d** (Extended Data Fig. 7 contains all clusters). **c**, Same UMAP embedding as in **b**, colored by the identity of the regulatory element promoter (TSS ± 2 kb) or distal (black), showing that bivalent peaks (H3K4me3/H3K27me3) as well as promoter peaks (H3K4me3/H3K27ac) and active peaks (H3K4me3) are enriched at promoters, whereas H3K27ac and H3K27me3 peaks are enriched at distal regulatory elements. **d**, Bar plots showing GREAT enriched terms for the clusters highlighted in **b** (Extended Data Fig. 7f). Categories concerning the nervous system, nervous system development or general development are colored. Unlabeled bars contain similar GREAT enriched terms as the bar above. Transcription factor motifs enriched per cluster are shown below each bar plot. snRNA proces., small nuclear RNA processing; Nerv. sys., nervous system; Postsyn., postsynaptic.

**Extended Data Fig. 7:**
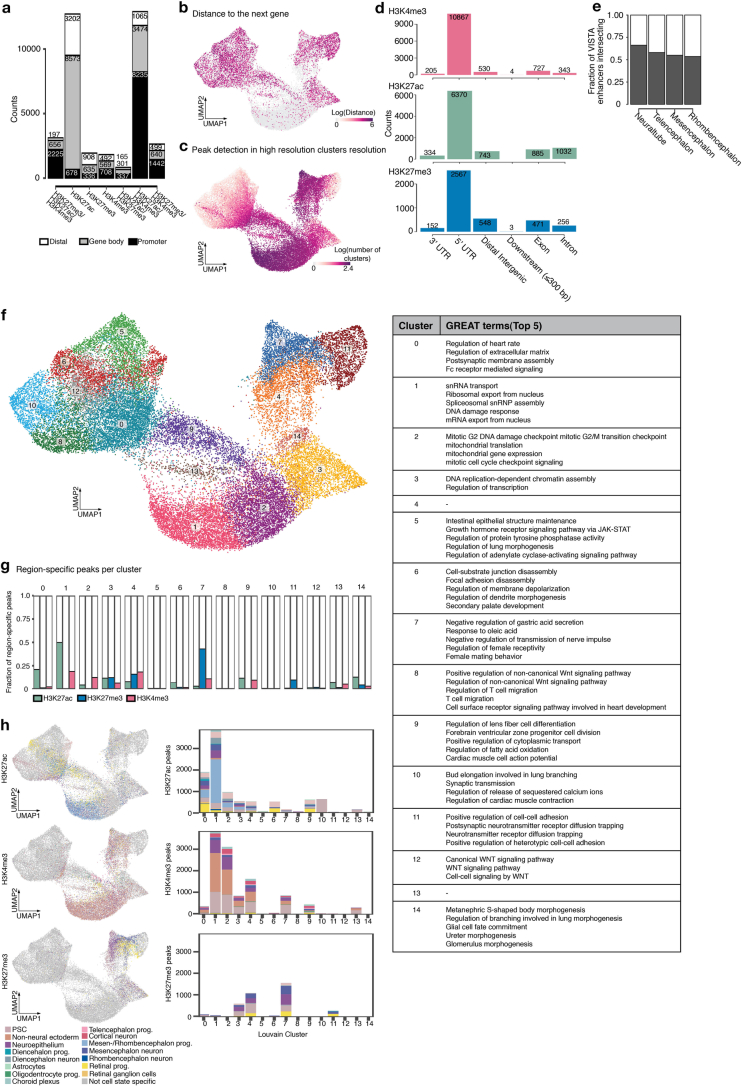
Characterization of regulatory elements within the developmental time course. **a**, Stacked barplot showing counts of distal, gene body and promoter peaks for all different classes of regulatory regions identified in the time course. **b**, UMAP embedding of all identified genomic regions from Fig. 3b colored by log10-transformed distance to the next gene. **c**, UMAP embedding of different peak classes colored by log10-transformed detection in high-resolution single-cell clusters. **d**, Barplots showing annotation of peaks in regulatory regions per histone modification (see Methods ‘Annotation of peak regions’). **e**, Barplot showing the fraction of validated VISTA enhancers for different brain regions intersecting with scCUT&Tag regulatory regions. **f**, UMAP embedding of regulatory regions from Fig. 3b colored by Louvain cluster identity (left panel). Table of top 5 significantly enriched terms in GREAT analysis (right panel) (see Supplementary Table 8 for more details). **g**, Barplot showing the fraction of brain region-specific peaks per cluster and histone modification. **h**, UMAP embedding of regulatory regions from Fig. 3b colored by the cell state where the respective peak is uniquely identified for each histone modification (left panel). Barplots quantifying the cell state specific peaks per Louvain cluster identified in f for each histone modification (right panel).

Next, we sought to characterize the different chromatin states within the dataset unbiasedly. We first applied rigorous filtering criteria to the peak set and performed dimensionality reduction and embedding using UMAP on all different classes of peaks based on their detection in high-resolution clusters (Fig. 3a,b). This clustering revealed distinct regulatory states of the different classes of histone modifications and separated promoter and distal regions (Fig. 3b,c and Extended Data Fig. 7a,b). Overall, promoter elements were detected in more high-resolution clusters than distal elements (Extended Data Fig. 7c). We quantified the enrichment of the different histone modifications across the genome and, as expected, found that all marks, and particularly H3K4me3, showed strong enrichment around the 5′ untranslated region (UTR) and promoter region, whereas H3K27ac and H3K27me3 showed enrichment also at distal intergenic sites (Extended Data Fig. 7d).

The peaks identified in this timecourse intersected with 50–60% of experimentally validated enhancers of the VISTA collection that vary across brain regions^34^, which suggests that the timecourse covers the majority of epigenetic states of brain region development (Extended Data Fig. 7e). To identify whether this representation of regulatory regions could also reveal co-regulation of peak sets, we performed cluster analysis and used Genomic Regions Enrichment of Annotations Tool (GREAT) enrichment^26^ to annotate the clusters (Fig. 3d, Extended Data Fig. 7f–h and Supplementary Tables 8 and 9). We found distinct enrichments for all different peak classes. Among the promoter clusters, we identified one cluster containing mostly housekeeping functions and nuclear processes (cluster 1), and another cluster contained peaks involved in nervous system development and specific neuronal processes (for example, generation of action potentials—cluster 2). This cluster also showed enrichment for neuronal transcription factors (POU4F2 and LMX1B). Distal elements enriched in H3K27ac (clusters 3 and 4) were also strongly enriched for terms and transcription factors related to neuronal, glia and nervous system development (NEUROD1, NEUROG2 and NFIB). For H3K27me3 peaks, we identified two major clusters. One contained more promoter peaks and showed co-enrichment for H3K4me3 (cluster 5, previously referred to as ‘bivalent’). This cluster of peaks was enriched for general developmental processes. Cluster 6 contained distal H3K27me3 enriched peaks and was enriched with terms important for neuron homeostasis and nerve development. We found that most peaks are not exclusive to one cell state but are dynamically marked by different histone modifications (active and repressive) in several different cell states (Extended Data Fig. 7f–h).

### Epigenetic activation precedes gene expression during neurogenesis

**Extended Data Fig. 8:**
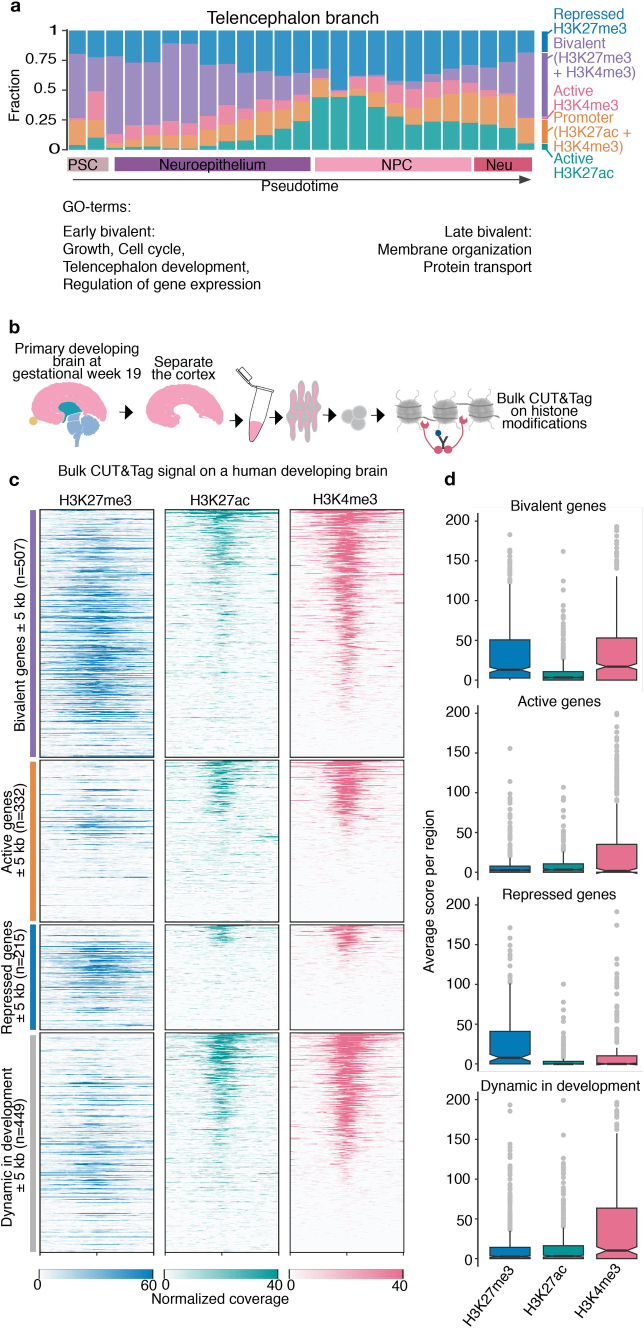
Chromatin states of the telencephalon branch in organoid and primary data. **a**, Stacked barplot showing the fraction of different chromatin states over pseudotime bins from PSC to neurons during brain organoid development (see Methods ‘Distribution of chromatin states during differentiation pseudotime’ for details). Bivalent domains are enriched in PSCs, depleted in NPCs and become abundant again at the neuronal stage. GO enrichment analysis of genes close to bivalent domains reveals enrichment for growth, cell cycle, telencephalon development, regulation of gene expression at early stages (PSC and neuroepithelium) and membrane organization and protein transport at later stages (NPC and neuron). **b**, Schematic of the experiment. The cortex was dissected from human developing brain samples and profiled using bulk CUT&Tag on H3K27me3, H3K27ac and H3K4me3 histone modifications. **c**, H3K27me3, H3K27ac and H3K4me3 bulk CUT&Tag signal from a primary developing cortex at gw 19 grouped by chromatin states identified from organoids in (**a**), (bivalent, active, repressed and dynamically changing). Region groups from (**a**) were identified starting with NPC to neurons. We plotted the signal on the TSS of the gene closest to the domain. For the categories active and repressed, only genes that were exclusively active/repressed in 90% of bins were considered**. d**, Boxplots showing the aggregated signal of the respective epigenetic marks for all region groups (n = 507 bivalent genes, n = 332 active genes, n = 215 repressed genes, n = 449 genes dynamically marked throughout development).

We next explored histone modification dynamics during differentiation from PSCs to region-specific neurons. Focusing on the dorsal telencephalon trajectory, we ordered cells along pseudotime using RNA velocity^22^ for RNA and diffusion maps^35^ for histone marks. To obtain an even timepoint distribution over pseudotime for all modalities, we subsampled cells before grouping them into 50 equally sized bins. As expected, we found that pluripotent cells are enriched in chromatin domains co-marked by repressive H3K27me3 and activating H3K4me3 (ref. ^8^) (Extended Data Fig. 8a), which decrease along with a gain of repressed (H3K27me3) and active (H3K27ac) domains until the NPC stage. Interestingly, H3K27me3 and H3K4me3 co-marked domains increase in abundance again at the end of the trajectory when NPCs differentiate into neurons (Extended Data Fig. 8a). We validated the detected signal with bulk CUT&Tag data from the human developing cortex (Extended Data Figs. 4b–f and 8b–d) and found that the chromatin status of repressed, active and H3K27me3/H3K4me3 co-marked domains was consistent with the organoid data. Due to the limitations of bulk measurements, we cannot rule out that some of the bivalency annotated in the primary human developing cortex data is attributed to tissue heterogeneity. However, the primary tissue mainly contained cells of the telencephalon lineage, and we found a complete overlap of H3K27me3 and H3K4me3 in 90% of the regions that we annotate as bivalent.

**Fig. 4:**
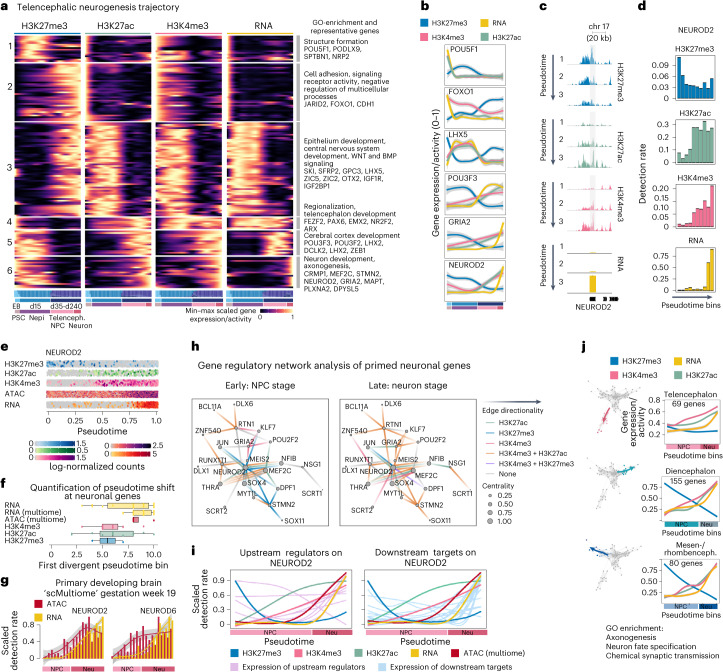
Pseudotime reconstruction from pluripotency reveals epigenetic priming during neuronal differentiation. **a**, Heatmap (left) showing gene activity scores for H3K27me3, H3K27ac and H3K4me3 as well as RNA expression over the telencephalic neuron differentiation trajectory from pluripotency (Methods). All *k*-means clusters are annotated with representative GO terms and example genes (right). Bar plots below show the timepoint distribution. Nepi, neuroepithelium. **b**, Line plots showing scaled expression and gene activity scores across pseudotime bins of selected examples from multiple *k*-means clusters (Methods). Genes expressed during pluripotency and at the progenitor stage show accurate alignment of the pseudotimes for RNA expression and active histone modifications (POU5F1, FOXO1 and LHX5), whereas neuron-specific genes exhibit epigenetic priming (GRIA2 and NEUROD2). **c**, Genome browser snapshots of the neuron-specific gene NEUROD2 showing levels of H3K27me3, H3K27ac and H3K4me3 on the gene and RNA expression over pseudotime. **d**, Quantification of histone modifications and RNA expression on the NEUROD2 locus (**c**) over pseudotime, showing chromatin regulation preceding RNA expression. **e**, Jitter plots showing log-normalized fragment counts along pseudotime. **f**, Quantification of the shift in pseudotime among the establishment of histone modifications, chromatin accessibility and RNA expression (*n* = 19 genes, median ± Q1/Q3; Methods). **g**, Detection rate of scATAC and scRNA expression measured from the same cells of a primary developing brain at 19 gw; the neuron-specific genes NEUROD2 and NEUROD6 show priming of chromatin (Supplementary Fig. 4g–i). Neu, neuron. **h**, GRN inferred using Pando from multiome data colored by regulatory regions detected in scCUT&Tag modalities (Methods). **i**, Scaled detection rate of NEUROD2 upstream regulators (pink—left; for example, HEY1, KLF7 and SOX2) and downstream targets (light blue—right; for example, CNTN1 and RAS11B) identified from the GRN over pseudotime. **j**, Force-directed layout of the telencephalon, diencephalon and mesen-/rhombencephalon branch (left). Line plots showing smoothed, averaged pseudotemporal gene expression and activities of *k*-means clusters with late pseudotime expression (Supplementary Fig. 6a–f) in the telencephalon (cluster 1), diencephalon (cluster 1) and mesen-/rhombencephalon (cluster 4) trajectory (right). Representative GO terms of genes within the *k*-means clusters with late pseudotime expression reveal, again, terms related to neuronal identity (Supplementary Tables 13–15).

Next, we clustered genes based on their pseudotemporal expression and histone modification patterns and found six major gene groups with distinct pseudotemporal dynamics (Fig. 4a; Supplementary Table 10 contains the full list of clusters). Clusters 1 and 2 (GO enrichment: structure formation, cell adhesion and signaling receptor activity) capture the epigenetic silencing of pluripotency genes during the transition from pluripotency to neuroepithelium (Fig. 4a) following two different pseudotemporal dynamics: genes in cluster 1 become downregulated and lose active histone modifications while gaining H3K27me3 at the neuroepithelium stage (Fig. 4b; POU5F1), whereas genes in cluster 2 install H3K27me3 only at the NPC stage while expression decreases right after exiting pluripotency (Fig. 4b; FOXO1). Cluster 3 genes (Fig. 4a; GO enrichment: epithelium and central nervous system development) are expressed in the neuroepithelium and in neural progenitors and show transcription reduction concurrently with gain in H3K27me3 (Fig. 4b; LHX5). In contrast, genes in clusters 4 and 5 (Fig. 4a; GO enrichment: regionalization, telencephalon development and cerebral cortex development) are also expressed in NPCs but showed an increase in active histone modifications and expression after H3K27me3 levels decreased (Fig. 4b; POU3F3). Strikingly, we observed that neuronal genes in cluster 6 lose H3K27me3-mediated repression and accumulate H3K27ac and H3K4me3 marks before RNA expression initiates (Fig. 4b–f; NEUROD2 and GRIA2).

To consolidate this analysis and ameliorate the influence of potential confounders, such as distribution of timepoints and data quality, we repeated the pseudotime inference for the single timepoint day 120 after strict quality filtering using diffusion maps for all modalities (Supplementary Fig. 3a–d). After clustering, we obtained, again, a cluster enriched in neuronal genes (Supplementary Fig. 3b, cluster 5, and Supplementary Table 11) that showed priming with activating marks before RNA expression.

To characterize the series of events leading to transcriptional activation of the primed genes, we measured transcriptome and chromatin accessibility in the same single cells (sc-Multiome-seq) using the single-cell suspension of 120-d organoids also used for scCUT&Tag. This revealed that the activating histone modifications H3K27ac and H3K4me3 are established shortly before an increase in chromatin accessibility can be detected, followed by RNA expression (Fig. 4e,f). We observed the same effect in unbiasedly selected neuronal genes (Supplementary Fig. 4a–c). These results support a model in which chromatin can be primed with activating histone modifications directing the future expression of target genes^1,36^.

We used our single-cell multiome data of the human developing brain (19 gw), where transcription and chromatin accessibility are recorded from the same cell, to test whether neuronal genes also show epigenetic priming in the primary human cortex. Indeed, neuronal genes (NEUROD2, NEUROD6, BCL11B and STMN2) showed a gain in accessibility before RNA expression in the developing human cortex (Fig. 4g and Supplementary Fig. 4d–i). This supports a role for epigenetic priming during nervous system development, in particular for neuron-specific genes^36,37^, and similar to reports from other developmental systems^1,6,38–40^. Recently, H3K4me3 was shown to be required for pausing release of PolII (ref. ^41^). It is interesting to hypothesize that H3K4me3 marks on these neuronal genes build up gradually before PolII is released into elongation.

To investigate the gene regulatory mechanisms underlying neuronal gene priming, we inferred a GRN based on an integrated scRNA-seq and scATAC-seq dataset of organoid development^14^ informed by histone modification-marked regulatory regions in our scCUT&Tag dataset. We focused on a subnetwork including all genes in the neuronal cluster as well as common transcription regulators that were shared between at least three of the genes in the neuronal cluster. We then summarized epigenetic modifications over pseudotime at transcription factor binding regions. This revealed bHLH transcription factors (such as NEUROD2 or NEUROG2; Supplementary Fig. 5a–e) as a central hub in this network and showed stepwise activation with loss of repressive marks and gain of active chromatin marks on its regulatory elements throughout pseudotime (Fig. 4h and Supplementary Table 12). In line with NEUROD2 being a central mediator of gene regulation in the primed cluster, bHLH transcription factors have been suggested to act as priming factors at lineage-primed enhancers^42^. We examined the expression of genes acting upstream and downstream of NEUROD2 and found that most upstream regulators (for example, HEY1, KLF7 and SOX2) are expressed after activating marks are installed and chromatin has gained accessibility at the NEUROD2 locus but before NEUROD2 is expressed (Fig. 4i, expression profiles in pink). This suggests that activating chromatin marks establish a transcriptionally permissive landscape before NEUROD2 transcriptional regulation. In contrast, and, as expected, most downstream target genes (such as CNTN1 and RAS11B, expression profiles in blue) are expressed after activation of NEUROD2 expression (Fig. 4i). To explore epigenetic priming of neuronal genes in other brain region trajectories, we extended the pseudotime analysis to the diencephalon and mesen-/rhombencephalon branch (Fig. 4j). We identified two clusters of late expressed neuronal genes in all branches (for example, KCNN2, MAP3K13, NEUROD2, SLIT1, STMN2, SLC6A1, NSG1 and CPLX2) for which we detected a similar epigenetic priming as observed in the telencephalon (Fig. 4a,j, Supplementary Fig. 6a–f and Supplementary Tables 13–15).

Taken together, our analysis provides insight into the dynamic interplay of histone modifications and RNA expression during differentiation from pluripotency to neurons in distinct regions of the developing human brain organoid and shows epigenetic priming of neuronal genes.

### Perturbation of H3K27me3 reveals its role during fate acquisition

**Fig. 5:**
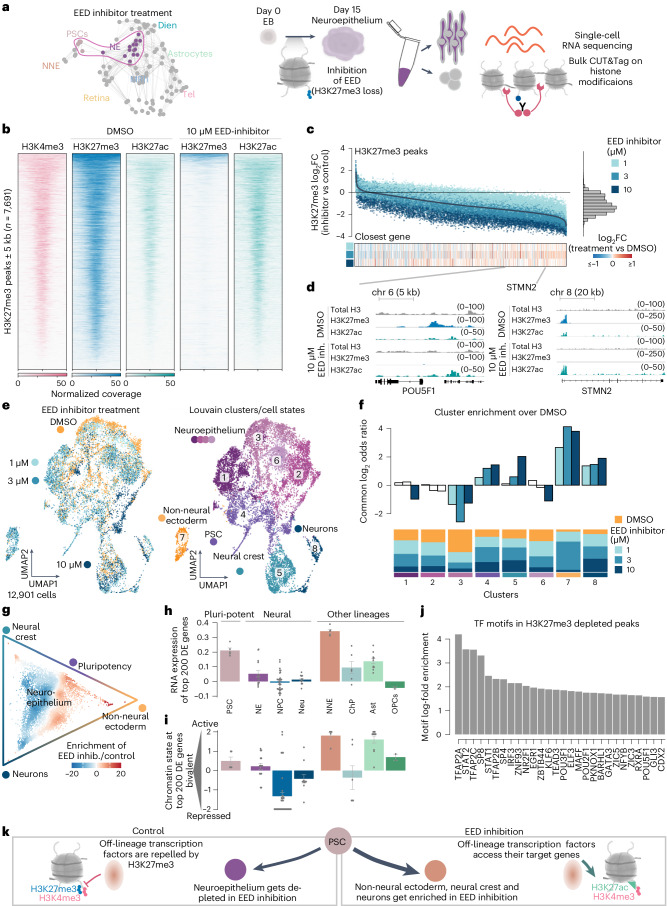
Aberrant cell fate acquisition upon H3K27me3 depletion. **a**, Schematic of the experiment. **b**, Heatmaps showing H3K4me3, H3K27me3 and H3K27ac bulk CUT&Tag signal on H3K27me3 peaks in control and H3K27me3 as well as H3K27ac signal upon EED inhibitor treatment. Regions are ordered by H3K27me3 intensity (two biological replicates). **c**, Scatter plot showing the log_2_ fold change of the CUT&Tag signal on H3K27me3 peaks in organoids treated with different EED inhibitor concentrations versus control (top) and histogram showing the distribution of fold changes (right). Heatmap showing the log_2_ fold change of the expression of the closest gene from the DE analysis in scRNA-seq data (bottom). **d**, Genomic tracks showing bulk CUT&Tag profiles for H3K27me3 and H3K27ac at genomic regions around STMN2 and POU5F1. **e**, UMAP embedding of the scRNA-seq data colored by treatment (left) and annotated Louvain cluster identity (right), 12,901 cells from two biological replicates, each pool containing 50 organoids. **f**, Bar plot showing cluster enrichment of treated cells versus control (top) and distribution of treatments in clusters (bottom). Common odds ratio (height of the bar) and *P* value (transparency of the bar) were obtained from a two-sided Cochran–Mantel–Haenszel test stratified by sampling timepoint (Methods). **g**, Circular plot of differential transition probabilities between the different cell states (defined in **e**) (Methods and Extended Data Fig. 9h). Inhibitor-treated cells show an enrichment at the terminal states of the graph. Cells tend to remain pluripotent or differentiate in neurons or off-lineage states, such as neural crest and NNE. **h**,**i**, Bar plots quantifying the expression (**h**) and bivalency (**i**) of the top 200 differentially expressed genes in the cell populations (*n* = 200 genes) identified from the developmental brain organoid atlas (Fig. 1c). Error bars denote the standard deviation. Genes with bivalent histone modifications tend to become upregulated in PSCs and NE (Extended Data Fig. 10c,d). **j**, Transcription factor motif enrichment in H3K27me3-depleted peaks close (10 kb) to differentially expressed genes in the NNE (cluster 7; Extended Data Fig. 10e,f). **k**, Schematic showing how H3K27me3-mediated repression of transcription factor motifs could affect lineage decisions when cells exit from pluripotency and lead to preferential stabilization of the NNE fate. NE, neuroepithelium.

**Extended Data Fig. 9:**
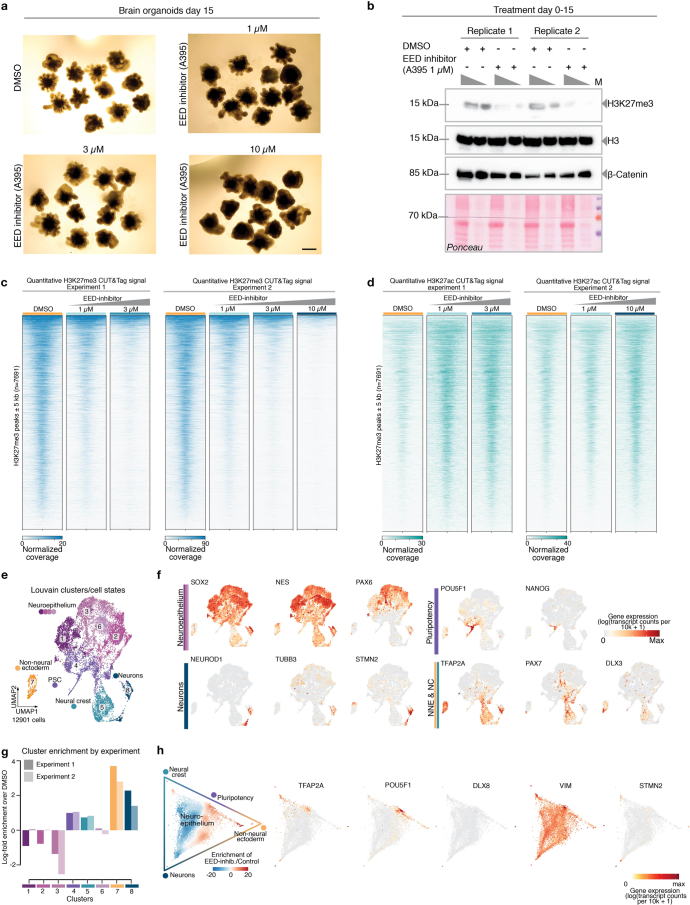
Aberrant cell fate acquisition upon depletion of H3K27me3. **a**, Brightfield images of organoids at day 15 treated with DMSO (control) or different concentrations of A395, an EED inhibitor. **b**, Western Blot of cellular extracts of 15 day old organoids shows depletion of H3K27me3 upon treatment with 1 µM A395. H3, β-Catenin and Ponceau serve as loading controls. This is a representative image of two replicates. The experiment has been performed 3 times on independent organoid batches. **c**, Heatmap of bulk H3K27me3 CUT&Tag signal on H3K27me3 peaks of two replicates of the inhibitor treatment ordered by intensity, showing consistent depletion of H3K27me3. **d**, Heatmap of bulk H3K27ac CUT&Tag signal on H3K27me3 peaks of two replicates of the inhibitor treatment ordered by intensity, showing consistent increase of H3K27ac. **e**, UMAP embedding colored by Louvain cluster annotation of the DMSO control and all inhibitor treated cells. **f**, UMAP embedding colored by expression of genes marking annotated Louvain clusters from (e). **g**, Barplot showing the log2 fold enrichment of different Louvain clusters over DMSO for the two replicates of the EED inhibitor treatment. Showing consistent trends between the experiments. **h**, Circular layout of differential transition probabilities between different cell states from the inhibitor treatment, showing an enrichment of H3K27me3 depleted cells at the terminal states of the graph (left), the circular plot colored by the expression of different marker genes (right) (see Methods ‘Inference of terminal fate probabilities in the perturbation experiment’ for details).

We tested the role of histone modifications in early brain organoid development by inhibiting H3K27me3 (EED/PRC2) with A395 43 from pluripotency until the neuroepithelium stage (day 0–15; Fig. 5a and Extended Data Fig. 9a,b), overcoming strong developmental defects in full PRC2 knockouts (KOs)^43,44^. At later stages, depletion of EZH2 mouse cortical progenitors altered timing and favored differentiation over self-renewal^45^. At the neuroepithelial stage (days 15–18), we profiled EED inhibitor–treated and control organoids using scRNA-seq and bulk CUT&Tag (Fig. 5a and Extended Data Fig. 9c). This revealed a concentration-dependent depletion of H3K27me3 and enrichment of the competitive, activating H3K27ac histone mark (Fig. 5b,c, Extended Data Fig. 9c,d and Supplementary Table 17). Many H3K27me3 peaks also showed co-enrichment for H3K4me3 in the neuroepithelium (Fig. 5b). By scRNA-seq, we observed an upregulation of gene expression in proximity to H3K27me3-depleted peaks (Fig. 5c and Supplementary Table 17). STMN2 and POU5F1 are exemplary genes showing strong H3K27me3 depletion, concomitant gain of H3K27ac and upregulation of mRNA expression (Fig. 5c,d).

**Extended Data Fig. 10:**
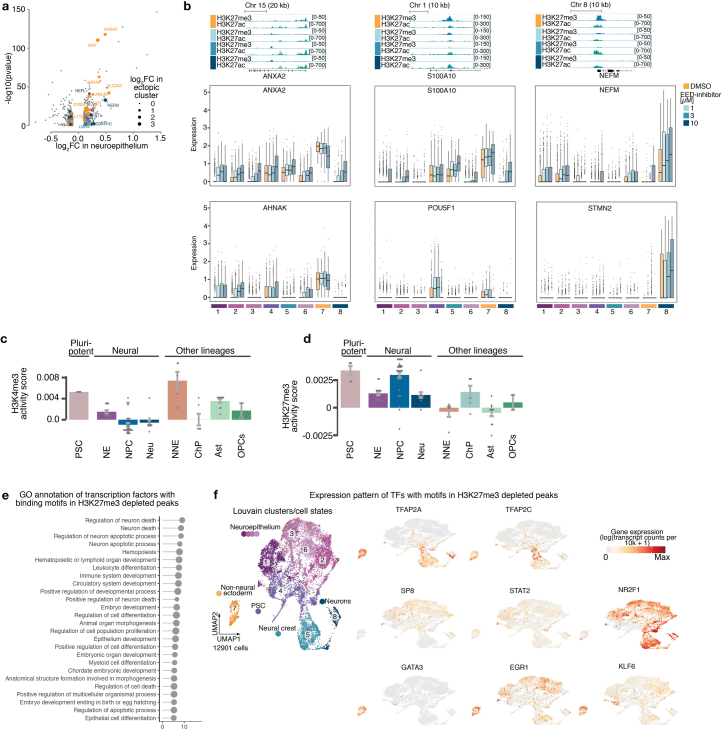
Effect of H3K27me3 depletion on lineage commitment in human brain organoids. **a**, Vulcano plot of the log2 fold change between the inhibitor treatment and the controls in the neuroepithelium clusters (1-4 and 6) versus the –log10(p-value). P-values were derived from a likelihood ratio test and were multiple testing corrected using the Benjamini-Hochberg method. Genes are colored by the identity of the ectopically formed clusters where they act as cluster markers (for example ANXA2, S100A10, non-neural ectoderm; STMN2, NEFM, neurons, see Methods Differential gene expression analysis in the perturbation experiment for details). **b**, IGV browser snapshot showing the inhibitor concentration dependent reduction of H3K27me3 and gain of H3K27ac at selected targets from (a) (top panel) Boxplot of upregulated genes in the neural epithelium showing gene expression per cluster (n = 12901 cells, bottom panel). **c**, Barplots quantifying the gene activity score of H3K4me3 on the top 200 DE genes between inhibitor treatment and control (n = 200 genes). **d**, same as c for H3K27me3. Error bars in c and d denote the standard deviation. **e**, GO term enrichment of transcription factors corresponding to enriched binding motifs in H3K27me3 depleted peaks in proximity to differentially genes non-neural ectoderm (cluster 7). **f**, UMAP embedding colored by cluster annotation of the DMSO control and all inhibitor treated cells (left panel). UMAP embedding colored by gene expression for several transcription factors with enriched binding motifs (Fig. 5j) in H3K27me3 depleted peaks close to deregulated genes (right panel).

We integrated the scRNA-seq data between conditions, performed clustering and annotated the resulting populations (Fig. 5e). We observed a large neuroepithelial progenitor population (clusters 1, 2, 3 and 6) but found inhibitor-treated cells enriched among pluripotent cells (cluster 4), NNE (cluster 7), neural crest (cluster 5) and neurons (cluster 8) (Fig. 5e,f and Extended Data Fig. 9e–g). We calculated transition probabilities using CellRank^22,23^ and confirmed that cells transition from the neuroepithelium and pluripotency to NNE, neural crest and neurons (Fig. 5g and Extended Data Fig. 9h). We performed differential expression (DE) analysis in the neuroepithelium cluster and found that many treatment-upregulated genes were also marker genes for the ectopic clusters (Extended Data Fig. 10a and Supplementary Tables 18–20). These genes exhibit an inhibitor-dependent loss of H3K27me3, gain in H3K27ac and upregulation of mRNA (Extended Data Fig. 10b), indicating that loss of H3K27me3 shifted cell identity toward ectopic cellular states. Indeed, the top upregulated genes upon H3K27me3 inhibition were expressed outside of the neuroepithelium in our developmental atlas (Fig. 5h). We found that genes most strongly upregulated upon loss of H3K27me3 resided in transcriptionally permissive H3K27me3/H3K4me3 bivalent domains within the neuroepithelium and PSCs, thereby primed for activation upon H3K27me3 loss (Fig. 5b,i). This suggests that H3K27me3 is required to stabilize cell fate decisions when cells progress from pluripotency to neuroepithelium (Fig. 5h,i and Extended Data Fig. 10c,d).

We investigated which transcription factors might destabilize cell fate decisions at the exit from pluripotency by analyzing binding motifs within H3K27me3-depleted peaks in close proximity to upregulated genes in the ectopic clusters (Fig. 5j). This revealed a motif enrichment for the TFAP2 family members that regulate the specification of neural crest and NNE^46,47^, STAT family members that are involved in cell proliferation and multiple transcription factors that regulate neuronal developmental processes (for example, EGR1, KLF6 and SP8) (Extended Data Fig. 10e). Most of them are expressed during the neuroepithelium stage (Extended Data Fig. 10f). We hypothesize that these transcription factors have enhanced access to their target genes upon depletion of H3K27me3 and induce a cascade of dysregulation that alters cell fate choice (Fig. 5k). Our observation offers a mechanistic explanation to the alterations in developmental timing observed in other systems^10,45,48^. Interestingly, approximately 60% of cells still established neuroepithelial identities, despite loss of H3K27me3. This supports that the neuroectoderm comprises a default fate that cells assume without inductive signals^49,50^.

Overall, we identified the molecular framework of how H3K27me3-mediated repression ensures lineage fidelity during early central nervous system development. We delineate its effect on immediate branch points within early developmental stages at single-cell resolution.

## Discussion

During early human brain development, gene expression must be tightly coordinated to enable controlled differentiation into various cell types. It has been challenging to study the epigenetic mechanisms that influence these dynamic processes on a global scale and with single-cell resolution. In our research, we addressed this by measuring histone modifications at single-cell resolution in brain organoids, which simulate critical aspects of early human brain regionalization and cell type formation in vitro. Throughout a developmental organoid timecourse, we combined scCUT&Tag profiles indicating repressed and active chromatin states with RNA expression data. We discovered that chromatin modification profiles were highly specific to particular cell populations, and we identified region-specific regulatory elements near key cell fate determinants, often marked by epigenetic switches at fate bifurcation points to prime gene expression. These findings suggest that chromatin modifiers are essential in regional diversification and cell fate stabilization, supported by EED perturbation experiments, which led to widespread H3K27me3 depletion, activation of typically repressed genes and cells adopting unintended states. These results imply that dynamic histone modifications are crucial for proper cell fate determination and brain regionalization. Additionally, our developmental atlas of histone marks will serve as a valuable reference for understanding the epigenomic landscape of human brain development, aiding future studies on cell fate commitment and reprogramming.

## Methods

The use of human embryonic stem (ES) cells for the generation of brain organoids was approved by the ethics committee of northwest and central Switzerland (2019-01016) and the Swiss federal office of public health. Under the Swiss Human Research Act, research performed with fully anonymized human specimens does not require an institutional review for research as long as consent was approved in the first place.

### Cell and organoid culture

Cell lines used in the study were derived from different sources:

409b2-iCRISPR (female), originally from the RIKEN BRC cell bank, modified by the S. Pääbo laboratory^51^

HOIK1 (female), HipSci resource^52^

WIBJ2 (female), HipSci resource^52^

WTC (GM25256) (male), Coriell Institute^53^

B7 (female), B. Roska laboratory^54^

For culturing, cells were grown on Matrigel (Corning, 354277) coated six-well dishes in mTeSR Plus (STEMCELL Technologies, 100-0276) supplemented with penicillin–streptomycin (1:200, Gibco, 15140122). To propagate the cells, they were dissociated with TryplE (Gibco, 12605010) or EDTA in DPBS (final concentration 0.5 mM) (Gibco, 15575020) and kept on Rho-associated protein kinase (ROCK) inhibitor Y-27632 (final concentration 5 μM, STEMCELL Technologies, 72302) for 1 d. Cells were stored in liquid nitrogen in mFreSR (STEMCELL Technologies, 05855) and tested for mycoplasma (Venor GeM Classic, Minerva Biolabs) after each thawing cycle.

To generate brain organoids, cells were grown to a confluency of approximately 50% and dissociated with TryplE. In total, 2,000–3,000 cells were aggregated in 96-well ultra-low attachment plates (Corning, CLS7007) to form embryoid bodies (EBs). We followed an unguided protocol to obtain brain organoids^55^, with a few modifications. EBs were aggregated and cultured in mTeSR Plus, and neural induction medium was added when the EBs had reached around 400–500 µm (usually day 5). Retinoic acid containing neural differentiation medium was added only from day 40 onward^37^. Cerebral organoids were grown shaking in 6-cm dishes for up to 8 months. To generate retinal organoids, we applied a protocol that allows the simultaneous aggregation of hundreds of EBs in agarose molds^54^. After neural induction, these are transferred (1 week) to Matrigel-coated six-well plates and allowed to attach. The neural induction medium used in both protocols is highly similar, allowing comparability of the neuroepithelium stage (both media contain DMEM/F12 (Gibco, 31331‒028), 1× N2 supplement (Gibco, 17502‒048), 1% NEAA solution (Sigma-Aldrich, M7145) and 2 mg ml^−1^ heparin (Sigma-Aldrich, H3149‒50KU) (1 µl ml^−1^ for brain organoids); in case of brain organoids, 1% GlutaMAX was added). After 2 weeks, neural differentiation medium was added, and retinal structures were scraped off after 4 weeks.

### Drug treatment

We targeted the H3K27me3-reader EED^56^ (A395, MedChemExpress, HY-101512) that prevents allosteric activation of the PRC2 complex. For A395, concentrations between 1 µM and 10 µM were tested, and H3K27me3 was depleted in bulk experiments at 1 µM as shown by western blot. EED was inhibited from days 0–15 (experiment 1) and days 0–18 (experiment 2), by adding the inhibitor when seeding the EBs. This time window coincides with the formation of the neural epithelium. The medium was changed every other day, and a fresh dose of inhibitors was addded. We treated two different cell lines (HOIK1 and WIBJ2). We used single-nucleotide polymorphisms (SNPs) to assign the cells bioinformatically to each cell line. Full blinding of the experiments on inhibitor-treated organoids was not possible due to the phenotypes evident from the development of organoids.

### Preparation of single-cell suspensions

Brain organoids were generated in batches. In each batch, five different stem cell lines (409b2-iCRISPR, B7, WTC, HOIK1 and WIBJ2) were used and dissociated together. The cell lines were demultiplexed using SNPs. For retinal organoids, only B7 was used. We sampled developmental transitions from the pluripotent EB (day 5) and neuroepithelium (day 15) to neuronal differentiation in brain (days 35, 60, 120 and 240) and in retina organoids (days 45 and 85). Organoids were cut into pieces using a scalpel and thoroughly washed with HBSS buffer without Ca^2+^/Mg^2+^ (STEMCELL Technologies, 37250).

A papain-based neural dissociation kit (Miltenyi Biotec, 130-092-628) was used to obtain single-cell suspensions. Then, 1,900 µl of pre-warmed buffer X with 50 µl of Enzyme P was added to the organoids and incubated for 15 min at 37 °C. A mix of 20 µl of buffer Y and 10 µl of DNase was added to each sample before tituration (10 times with a p1000 wide bore tip). The samples were then incubated twice for another 10 min and titurated with a p1000 and a p200 in between.

The reaction was stopped with HBSS buffer without Ca^2+^/Mg^2+^, and the cells were filtered at 30 µm. After an additional wash, the cells were stored in CryoStor CS10 (STEMCELL Technologies, 07930) or processed for further experiments. For all scCUT&Tag experiments, cells were kept from the same cell suspension to perform scRNA-seq. Cell viabilities were between 80% and 95%.

We performed the data collection on independent organoid batches from different cell lines. We demultiplexed them based on SNPs and treated them, therefore, as replicates. For day 60, we processed independent biological replicates, and, for day 120, we used the same cell suspension as a technical replicate. An overview of all the experiments is shown in Extended Data Fig. 1c. No statistical methods were used to pre-determine sample sizes, but our sample sizes are similar to those reported in previous publications^17–19^. No data points were excluded from the analysis. Organoids for each timepoint were picked blinded. First computational analysis and inspection of the data were performed blinded.

### Preparation of single-cell suspensions from human fetal brain

Human fetal brain tissue (19 gw) was obtained after elective pregnancy termination and informed written maternal consent from Advanced Bioscience Resources. The donor consented to the research use of the tissue without restrictions. The tissue was fully anonymized, and all experiments were performed in accordance with relevant guidelines and regulations. The estimated age of the fetus was calculated using clinical information, such as the last menstrual period and anatomical data obtained through ultrasound measurements. Dissected fetal brains were kept in DMEM + antibiotics on wet ice until preparation of the single-cell suspension for up to 48 h. Single-cell suspensions were prepared following the same protocol as for organoids. At this developmental timepoint, the cortex has already expanded and comprises the majority of the cells in the population. We tried to enrich cortical material by separating larger pieces from the material. The resulting single-cell suspensions were cryopreserved until further use. Nuclei suspensions for 10x multiome experiments were prepared following the scCUT&Tag protocol, and the nuclei were processed following the manufacturers’ instructions.

### Cloning and purification of Tn5

Plasmids no. 123461 (pA/G-MNase) and no. 124601 (3XFlag-pA-Tn5-Fl) were ordered from Addgene. Protein A and Protein G were amplified using the primer pairs (FZ461_ProtA_rev, FZ462_HindIII_ProtA_fw and FZ459_EcoRI_ProtG_rev, FZ460_ProtG_fw) and fused by polymerase chain rection (PCR) (below). Protein A in the original vector (3×Flag-pA-Tn5-Fl, Addgene, 124601 (ref. ^57^)) was then replaced with the fusion product through EcoRI and HindIII restriction digest.

The final plasmid was transformed into chemically competent Rosetta cells to express the protein. The bacteria were grown to an optical density at 600 nm (OD_600_) of 0.4–0.6; expression was induced with 0.25 mM IPTG; and the protein was expressed at 18 °C overnight. Cells were harvested and stored at −80 °C until further processing. The purification was performed on Chitin resin (New England Biolabs, S6651S) as described^58^, with small modifications. The cells were lysed using the Diagenode Bioraptor Plus at the high setting for 15 cycles, 30 s on/30 s off.

After dialysis and concentration using Amicon Ultra-4 Centrifugal filters (Millipore, UFC803024), the protein was diluted to 50% glycerol final and loaded with adapters before use (below).

FZ459_EcoRI_ProtG_revgaattctttatcgtcatctacggctggcgtcaactcagacgcg

FZ460_ProtG_fwaaaaagctaaacgatgctcaagcaccaaaaacaacttataaattagtcatcaacggg

FZ461_ProtA_revaatttataagttgtttttggtgcttgagcatcgtttagctttttagcttctgc

FZ462_HindIII_ProtA_fwccaagcttaaaagatgacccaagccaaagtgctaacc

FZ444_Tn5MErev[phos]CTGTCTCTTATACACATCT

FZ445_Tn5ME-ATCGTCGGCAGCGTCAGATGTGTATAAGAGACAG

FZ445_Tn5ME-BGTCTCGTGGGCTCGGAGATGTGTATAAGAGACAG

### scCUT&Tag

Starting from 1.5–3 Mio cells, nuclei were isolated following the 10x Genomics CG000365 Demonstrated Protocol. The 0.1× buffer was used for all experiments, adjusting the final concentration of Digitonin to 0.01% (Thermo Fisher Scientific, BN2006). In general, we followed the bulk CUT&Tag protocol, with a few adjustments^57^.

After lysis, the nuclei were directly transferred into scCUT&Tag wash buffer (20 mM HEPES (pH 7.5) (Jena Bioscience, CSS-511), 150 mM NaCl (Sigma-Aldrich, S6546), 0.5 mM Spermidine (Sigma-Aldrich, S0266), 1% BSA (Miltenyi Biotec, 30-091-376), 1 mM DTT (Sigma-Aldrich, 10197777001), 5 mM sodium butyrate (Sigma-Aldrich, 303410), Roche Protease Inhibitor (Sigma-Aldrich, 11873580001)) and washed once for 3 min at 300*g*. The buffer was supplemented with 2 mM EDTA before the antibodies were added (see Supplementary Table 21 for further details). The samples were incubated on a rocking platform at 4 °C overnight.

The next morning, the cells were washed once, and the secondary antibody was added to the suspension for 1 h at 20 °C on a rocking platform or Eppendorf thermomixer. The cells were then washed twice and transferred into scCUT&Tag med buffer (20 mM HEPES (pH 7.5) (Jena Bioscience, CSS-511), 300 mM NaCl (Sigma-Aldrich, S6546), 0.5 mM Spermidine (Sigma-Aldrich, S0266), 1% BSA (Miltenyi Biotec, 130-091-376), 1 mM DTT (Sigma-Aldrich, 10197777001), 5 mM sodium butyrate (Sigma-Aldrich, 303410), Roche Protease Inhibitor (Sigma-Aldrich, 11873580001)) including 2 µg of homemade Tn5. The cells were incubated for 1 h at 20 °C and then washed again twice with scCUT&Tag med buffer. To induce the cutting of the Tn5, 10 mM final MgCl_2_ (Sigma-Aldrich, M1028) was added, and the sample was incubated for 1 h at 37 °C. After the incubation, the reaction was stopped with 15 mM EDTA final, and the sample was filled up to 600 µl with diluted nuclei buffer of the 10x Genomics scATAC kit v1.1 supplemented with 2% BSA. The nuclei were filtered through a 40-µm Flowmi filter (Sigma-Aldrich, BAH136800040) and washed with diluted nuclei buffer supplemented with 2% BSA.

The final nuclei suspension was quality controlled and counted with a trypan blue assay on the automated cell counter Countess (Thermo Fisher Scientific). Finally, 15,000–20,000 nuclei were loaded per experiment. In cases where fewer nuclei were recovered, all nuclei were loaded. To run the Chromium Chip, 5 µl of cell suspension was mixed with 3 µl of PBS and 7 µl of ATAC buffer from the kit.

Libraries were prepared following the manufacturer’s instructions, except that two PCR cycles were added to the barcoding PCR, and, after 10 cycles of indexing PCR, 5 µl of the library was used to determine the final number of cycles in a Roche LightCycler^59^. Usually, scCUT&Tag libraries required 12–16 PCR cycles during the indexing. After SPRIselect clean-up (Beckman Coulter, B23318), the libraries were quality controlled and sequenced following the 10x Genomics scATAC v1.1 sequencing recommendations. Usually, 50–100 Mio reads per library were enough to cover the complexity.

H3K27me3 rabbit, Diagenode, C15410195, A0824D

H3K27ac rabbit, Diagenode, C15410196, A1723-0041D

H3K27ac mouse, monoclonal (MABI0309), GeneTex, GTX50903

H3K4me3 rabbit, Diagenode, C15410003, A1052D

H3 mouse, Active Motif, 39763, 20418023

β-Catenin mouse, 1:5,000, BD Biosciences, 610154

Guinea pig anti-rabbit antibodies online, ABIN101961

Alexa Fluor–conjugated antibodies, Thermo Fisher Scientific

HRP-conjugated antibodies, Jackson ImmunoResearch

### Bulk CUT&Tag

Starting with 0.1–1 Mio cells after dissociation, cells were transferred into CUT&Tag wash buffer (20 mM HEPES (pH 7.5) (Jena Bioscience, CSS-511), 150 mM NaCl (Sigma-Aldrich, S6546), 0.5 mM Spermidine (Sigma-Aldrich, S0266), 5 mM sodium butyrate (Sigma-Aldrich, 303410), Roche Protease Inhibitor (Sigma-Aldrich, 11873580001)). After 15 µl of BioMag, Concanavalin A beads (Polysciences, 86057-3) in binding buffer (20 mM HEPES (pH 7.5), 10 mM KCl, 1 mM CaCl_2_, 1 mM MnCl_2_) were added to the sample and incubated on the wheel for 15 min at room temperature. Subsequently, the cells were collected on a magnet and lysed through the addition of CUT&Tag wash buffer supplemented with 0.01% Digitonin. Lysis was monitored under a microscope with trypan blue staining. After lysis was complete, the nuclei were washed again with CUT&Tag wash buffer. If possible, all samples were split, and H3 or another chromatin mark CUT&Tag was performed on the same starting material, to be used as normalizer. The antibody was added together with 2 mM EDTA final, and the sample was incubated on a rocking platform at 4 °C overnight.

The samples were washed once with CUT&Tag wash buffer, and the secondary antibody was added to the reaction and incubated for 1 h at 20 °C on a rocking platform. After two additional washes, the Tn5 was added (1:100) in CUT&Tag med buffer (20 mM HEPES (pH 7.5) (Jena Bioscience, CSS-511), 300 mM NaCl (Sigma-Aldrich, S6546), 0.5 mM Spermidine (Sigma-Aldrich, S0266), 5 mM sodium butyrate (Sigma-Aldrich, 303410), Roche Protease Inhibitor (Sigma-Aldrich, 11873580001)). Tn5 was allowed to bind for 1 h at 20 °C on a rocking platform. After two additional washes, the cutting was induced through the addition of 10 mM MgCl_2_ in CUT&Tag med buffer. After 1 h at 37 °C, the reaction was stopped by adding a final of 20 mM EDTA, 0.5% SDS and 10 mg of Proteinase K. The reaction was then incubated at 55 °C for 30 min and finally inactivated at 70 °C for 20 min.

The DNA fragments were purified using the ChIP DNA Clean & Concentrator Kit (Zymo Research, D5205). For the elution from the columns 2 pg of Tn5-digested and purified lambda DNA (New England Biolabs, N3011S) were added to be used as spike-in normalizer for later analysis, when needed. Purified fragments were indexed for 15 cycles (1 × 5 min at 58 °C, 1 × 5 min at 72 °C, 1 × 30 s at 98 °C, 14 × 10 s at 98 °C, 30 s at 63 °C, 1 × 1 min at 72 °C, ∞ at 4 °C) using NEBNext HighFidelty 2× PCR Master Mix (New England Biolabs, M0541S) and Illumina i5 and i7 indices^59^. The libraries were then purified using AMPure beads (Beckman Coulter, A63881), measured and quality controlled with Qubit DNA HS Assay (Thermo Fisher Scientific, Q32854) and on a TapeStation (Agilent, 5067-4626) and then sequenced (PE, 2 × 50 bp).

### Hashing and scRNA-seq

Cells were either processed right after dissociation or recovered after cryostorage. To recover the cells after cryostorage, the cryovials were incubated in a water bath at 37 °C until only a small ice piece was left inside the tube. The cells were then transferred into pre-warmed DMEM/F-12 (Gibco, 31330038) supplemented with 10% FBS final (Merck, ES-009-B). After washing twice with DPBS (Gibco, 14190144) supplemented with 0.5% BSA, the cells were filtered with a 40-µm Flowmi filter and counted using the trypan blue assay on the automated cell counter Countess (Thermo Fisher Scientific). Cell viability was approximately 80–95%. Cells were further diluted to be processed in the 10x Genomics single-cell RNA expression v3.1 assay, strictly following the manufacturer’s guidelines.

To generate single-cell RNA expression libraries of the drug treatment, we turned to cell hashing^60^ for better comparability of the samples. For hashing, 100,000–300,000 cells were resuspended in 50–100 µl of DPBS + 0.5% BSA. Then, 5 µl of Human TruStain FcX (Fc Receptor Blocking Solution, BioLegend, 422302) was added to the sample and incubated for 10 min on ice. Next, 2 µl of TotalSeq Cell hashing antibodies (BioLegend) was added to the cells and incubated for 30 min on ice with gentle agitation every 10 min.

Replicate 1:

HTO1 - GTCAACTCTTTAGCG - DMSO_ctrl

HTO3 - TTCCGCCTCTCTTTG - 1 µM A395

HTO4 - AGTAAGTTCAGCGTA - 3 µM A395

Replicate 2:

HTO1 - GTCAACTCTTTAGCG - DMSO_ctrl

HTO5 - AAGTATCGTTTCGCA - 1 µM A395

HTO7 - TGTCTTTCCTGCCAG - 3 µM A395

HTO8 - CTCCTCTGCAATTAC - 10 µM A395

After incubation, cells were washed twice with DBPS + 0.5% BSA. Depending on the starting amount, the cells were resuspended in 20–40 µl of DPBS + 0.5% BSA and counted. Then, the cell suspensions were mixed and processed in the 10x Genomics single-cell RNA expression v3.1 assay, strictly following the manufacturer’s guidelines with slight adjustments. A maximum of 20,000 cells were targeted per experiment, and HTO additive primer was added to the cDNA synthesis following the TotalSeq technical protocol (https://www.biolegend.com/en-us/protocols/totalseq-a-antibodies-and-cell-hashing-with-10x-single-cell-3-reagent-kit-v3-3-1-protocol). To generate gene expression and hashing libraries, we followed the protocol CG000206 Chromium Next GEM SingleCell v3.1 Cell Surface Protein and sequenced according to the manufacturer’s guidelines.

### Western blot

Next, 1–3 organoids were directly collected into 50 µl of Laemmli sample buffer and homogenized with an electric grinder (Fisherbrand, 12-141-368). DNA was sheared by sonication in the Diagenode Bioraptor Plus (high setting for 15 cycles, 30 s on/30 s off). Samples were subsequently run on SDS-PAGE and transferred to PVDF membrane using Wet-Blot. Then, 2–10 µl of extract was loaded per lane. The ECL signal was recorded using the iBright system (Invitrogen). The signal was compared to antibody stainings of loading controls (H3 and β-Catenin), and the membranes were quality controlled by Ponceau (Sigma-Aldrich, P7170-1L) staining. See Supplementary Table 21 for details on the antibodies.

### Immunostaining

Organoids were fixed overnight in 4% paraformaldehyde. The next day, the organoids were washed three times for 5 min with DPBS and then transferred into 30% sucrose in DPBS until they sank to the bottom of the tube. Then, the organoids were transferred into cryomolds (Sakura, 4565) and embedded in Tissue-Tek O.C.T. (Sakura, 16-004004) on dry ice. The organoids were sliced on a Cryostar NX70 (Thermo Fisher Scientific) into 20-µm-thick slices at −17 °C. The slices were transferred to glass slides and washed with PBS. After a quick wash, antigen retrieval was performed for 20 min at 70 °C in 1× pre-heated HistoVT One (Nacalai, 06380). Slides were washed three times for 5 min with PBS + 0.2% Tween and then transferred to blocking and permeabilization (PBS, 0.1% Triton, 5% serum, 0.2% Tween, 0.5% BSA) for 1 h. The antibodies were added to the blocking solution overnight at 4 °C (see Supplementary Table 21 for further details). The next day, the slides were washed again three times for 5 min with PBS + 0.2% Tween, and the secondary antibody was added in PBS supplemented with 2% BSA and 0.2% Tween for 2 h at room temperature. Last, the slides were washed again three times for 5 min with PBS + 0.2% Tween, adding DAPI to the last wash. The slides were then mounted in Prolong Glass Antifade and imaged on a Nikon Ti2 spinning disk or a confocal Zeiss LSM 980 microscope.

### Data processing for scRNA-seq

To compute transcript count matrices, sequencing reads were aligned to the human genome and transcriptome (hg38, provided by 10x Genomics) by running Cell Ranger (version 5.0.0) with default parameters. Count matrices were then pre-processed using the Seurat R package (version 3.2)^61^.

Cells were filtered by unique molecular identifier (UMI), number of detected genes and fraction of mitochondrial genes as follows:

UMIs > 2,000

UMIs < 1.5 × 10^5^

detected genes > 1,000

fraction of mitochondrial reads < 0.2

Transcript counts were normalized by the total number of counts for that cell, multiplied by a scaling factor of 10,000 and subsequently natural-log transformed (NormalizeData()).

### Pre-processing and clustering of scCUT&Tag data

We aligned the sequencing reads to the human genome and transcriptome (hg38, provided by 10x Genomics) using Cell Ranger ATAC (version 1.2.0) with default parameters to obtain fragment files and peak calls. The fragment files and the peak count matrices were further pre-processed using Seurat (version 3.2)^62^ and Signac (version 1.1)^61^. We removed cells with fewer than 200 (H3K27ac and H3K4me3) or 100 (H3K27me3) fragment counts from the analysis. For quality control, we checked the following metrics using Signac: the transcription start site (TSS) enrichment score (TSSEnrichment()), in particular for activating and promoter marks; the nucleosome signal (NucleosomeSignal()); the percentage of reads in peaks; and the ratio of reads in genomic blacklist regions.

We then created a unified set of peaks from the union of peaks from all samples by merging overlapping and adjacent peaks. The unified set of peaks was re-quantified for each sample using the fragment file (FeatureMatrix()). Peak counts were normalized by term frequency-inverse document frequency (tf-idf) normalization using the Signac functions RunTFIDF(). Latent semantic indexing (LSI) was performed by running SVD (RunSVD()) on the tf-idf-normalized matrix. To visualize the data in two dimensions (2D), UMAP^12^ was performed on LSI components 2–30. We then called high-resolution clusters using Louvain clustering in each group separately with the following resolutions to obtain similar cluster sizes:

EB: 2

Mid: 5

Late: 10

8 months: 5

Retina: 5

### Demultiplexing

We used demuxlet^63^ to demultiplex cells pooled from different stem cell lines. For B7 and 409B2-iCRISPR, SNPs were called using bcftools based on DNA sequencing^52,64^ data or downloaded from the HipSci website (HOIK1 and WIBJ2) and the Allen Cell Atlas (WTC). All files were merged using bcftools, and sites with the same genotypes in all samples were filtered out. Demuxlet was run with default settings. For RNA, cells with ambiguous or doublet assignments were removed from the data. Otherwise, the best singlet assignment was considered the lines’ genotype.

### Integration and annotation of scRNA-seq data

First, we grouped the dataset into five groups depending on the sample of origin:

EB: 4-day-old brain organoids in EB stage

Mid: 15-day-old brain organoids in neuroepithelium stage

Late: 1–4-month-old brain organoids

8 months: 8-month-old brain organoids

Retina: 6-week-old and 12-week-old retina organoids

Initial integration was based on mid and late groups. We computed the 2,000 most variable features using the Seurat function FindVariableFeatures() and computed cell cycle scores using the Seurat function CellCycleScoring(). Subsequently, the data were z-scaled; cell cycle scores were regressed out (ScaleData()); and principal component analysis (PCA) was performed using the Seurat function RunPCA() based on variable features. We used the first 10 principal components (PCs) to integrate the different timepoints in the dataset using the Cluster Similarity Spectrum (CSS) method^65^. The missing samples EB, retina and 8 months were then projected into CSS space using the css_project() function. To obtain a 2D representation of the data, we performed UMAP^12^ using RunUMAP() with spread = 0.8, min.dist = 0.2 and otherwise default parameters.

To annotate the data, we first called high-resolution clusters using Louvain clustering in each group separately with the following resolutions to obtain similar cluster sizes:

EB: 1

Mid: 2

Late: 5

8 months: 2

Retina: 2

Clusters were annotated with cell types and regional identities using VoxHunt^66^, comparison to reference datasets^13,14^ and marker expression.

### Hashing

Count matrices were generated with CITE-seq-Count (version 1.4.5 running under Python version 3.6.13) and intersected with transcript matrices from Cell Ranger. The hashtag counts were normalized with centered log-ratio (CLR) transformation. Doublets were filtered out using hashtag information.

### Calculation of gene activity scores

To enable comparison of gene expression with chromatin modifications in the same feature space, we computed gene activity scores for each gene and chromatin modality. We summarized fragment counts over the gene body plus an extended promoter region (+2 kb). For this, we used the Signac function GeneActivity() with default parameters. Fragment counts representing gene activities were subsequently log normalized with a scaling factor of 10,000.

### Annotation of peak regions

To obtain genomic annotations for peak regions from all chromatin modalities, we used the function annotatePeak from the R package CHIPseeker^67^ with default parameters.

### Matching of scRNA and scCUT&Tag

To integrate cell populations between RNA and chromatin modalities, we matched high-resolution clusters based on the correlation of gene expression with gene activity scores. For this, we performed MCMF bipartite matching between the modalities as described in https://github.com/ratschlab/scim (ref. ^21^). The function get_cost_knn_graph() was used with knn_k = 10, null_cost_percentile = 99 and capacity_method = ‘uniform’. As a distance metric (knn_metric), we used the correlation distance provided by scipy^68^ for activating marks H3K27ac and H3K4me3 and the negative correlation distance for the repressive mark H3K27me3. Unmatched clusters from either modality were matched based on maximum (or minimum) correlation.

### Data representation

In all box plots, the center line denotes the median; boxes denote lower and upper quartiles (Q1 and Q3, respectively); whiskers denote 1.5× the interquartile region below Q1 and above Q3; and points denote outliers. All error bars shown in the manuscript depict the standard deviation. For bar plots, the absolute numbers are given within the plot or the legend.

### Graph representation of regional diversification

To visualize differentiation trajectories into regionalized neuronal populations, we constructed a graph representation based on terminal fate probabilities. For this, we first obtained count matrices for the spliced and unspliced transcriptome using kallisto (version 0.46.0)^69^ by running the command line tool loompy fromfastq from the Python package loompy (version 3.0.6)(https://linnarssonlab.org/loompy/). We subset the dataset to cell populations on the neuronal trajectory from pluripotency (PSCs, neuroepithelium, NPCs and neurons) and computed RNA velocity using scVelo (version 0.2.4)^22^ and scanpy (version 1.8.2)^70^. First, 2,000 highly variable features were selected using the function scanpy.pp.highly_variable_genes(). Subsequently, moments were computed in CSS space using the function scvelo.pp.moments() with n_neighbors = 20. RNA velocity was calculated using the function scvelo.tl.velocity() with mode = ‘stochastic’, and a velocity graph was constructed using scvelo.tl.velocity_graph() with default parameters. To order cells in the developmental trajectory, a root cell was chosen randomly from cells of the first timepoint (EB), and velocity pseudotime was computed with scvelo.tl.velocity_pseudotime(). The obtained velocity pseudotime was further rank transformed and divided by the total number of cells in the dataset. Based on the velocity pseudotime, we computed fate probabilities into the following manually annotated terminal cell states: dorsal telencephalon neurons, diencephalon neurons, midbrain neurons, hindbrain neurons and retinal ganglion cells. For this, we used CellRank (version 1.3.0)^23^. A transition matrix was constructed with a palantir kernel (PalantirKernel()) based on velocity pseudotime. Absorption probabilities for each of the pre-defined terminal states were computed using the GPCCA estimator. Based on the computed fate probabilities, we next constructed a graph representation. We used PAGA to compute the connectivities between clusters (scvelo.tl.paga()) and summarized transition scores for each of the clusters. To find branch points at which the transition probabilities into different fates diverge, we constructed a nearest-neighbor graph between the high-resolution clusters based on their transition scores (*k* = 10). We further pruned the graph to only retain edges going forward in pseudotime—that is, from a node with a lower velocity pseudotime to a node with a higher velocity pseudotime. Additionally, we removed edges connecting different regional trajectories. The resulting graph is directed with respect to pseudotemporal progression and represents a coarse-grained abstraction of the fate trajectory, connecting groups of cells with both similar transition probabilities to the different trajectories and high connectivities on the transcriptomic manifold.

### Differential peak analysis between regional identities

To find peaks with differential enrichment in regional trajectories, we performed differential peak analysis for each chromatin modality. We fit a GLN with binomial noise and logit link for each peak *i* on binarized peak counts Y with the total number of fragments per cell and the region label as the independent variables:

Y~n_fragments+region

In addition, we fit a null model, where the region label was omitted:

Y~n_fragments

We then used a likelihood ratio test to compare the goodness of fit of the two models using the lmtest R package (version 0.9) (https://cran.r-project.org/web/packages/lmtest/index.html). Multiple testing correction was performed using the Benjamini–Hochberg method.

### Analysis of bivalent and switching peaks

To find genomic regions that were marked by both H3K27me3 and H3K4me3 (bivalency), we extended all peaks of both modalities by 2 kb in both directions before intersecting them. For all intersecting peaks, we aimed to find instances where bivalency is resolved during regional diversification. For this, we selected matching peaks that showed differential enrichment in any region with opposite effect sizes. Out of these, we further selected matching peaks where both modalities were detected in more than 5% of cells in any high-resolution cluster in the neuroepithelial stage (bivalent in neuroepithelium). We performed an analogous analysis for H3K27me3 and H3K27ac to find regions where switching occurs upon regional diversification. Here, we selected matching peaks from both modalities for which only one mark was detected in the neuroepithelial stage (>5% detection in any high-resolution cluster).

### Embedding and clustering of regulatory regions

From peaks detected in all chromatin modalities, we aimed to reveal and stratify regulatory regions with distinct detection patterns across the developmental timecourse. We first applied rigorous quality control to all peaks in all modalities and retained only peaks that were detected in more than 50 high-resolution clusters and detected in more than 10% of cells in at least one cluster. We then merged peaks from all modalities through intersection and represented them by the detection rate of peaks in high-resolution clusters from all individual modalities. This resulted in a region × cluster matrix, which was z-scaled, and the number of detected clusters was regressed out using the Seurat function ScaleData(). Next, we performed PCA and UMAP embeddings, and Louvain clustering was performed based on 20 PCs. The class of regions was determined through the combination of chromatin marks that were detected in this region across the entire dataset. Regions were labeled as ‘promoters’ if they were within 2 kb of the TSS of a gene and as ‘distal’ if they were farther than 3 kb from a gene body.

### Functional enrichment of regulatory regions

Our previous analyses revealed brain region-specific peaks (top 50 differentially enriched peaks per region) and distinct clusters or regulatory regions. To understand if clusters of regulatory regions captured distinct functional enrichments or transcription factor binding sites, we performed functional enrichment with GREAT as well as transcription factor motif enrichment. GREAT enrichment was performed using the R package rGREAT^71^. We performed the analysis using the local implantation with the ‘GO:BP’ gene set based on TxDB hg38 gene definitions (https://bioconductor.org/packages/release/data/annotation/html/TxDb.Hsapiens.UCSC.hg38.knownGene.html). For transcription factor motif enrichment, we first discovered motifs in each peak using motifmatchr (version 1.14)^72^ through the Signac function FindMotifs(). Next, we performed a two-sided Fisherʼs exact test to test for differential enrichment of peaks versus background. *P* values from both analysis were multiple testing corrected using the Benjamini–Hochberg method. As a background for both analyses, we used the combined set of regulatory regions that passed filtering criteria.

### Reconstruction of the telencephalic neuron differentiation trajectory from pluripotency

To reconstruct the differentiation trajectory leading up to telencephalic neurons in higher resolution, we first extracted all cells annotated as EB, neuroepithelium, telencephalic progenitors and neurons. We next sought to compute a pseudotime describing the progression along this trajectory for all modalities separately. For all chromatin modalities, we used LSI components 2–10 to compute diffusion maps with the R package destiny^35^. Ranks along the first diffusion component were used as a pseudotemoral ordering. For RNA, we used the function scvelo.tl.velocity_pseudotime() from scVelo^22^ to compute a pseudotime based on RNA velocity. To obtain an even distribution of timepoints for all modalities, we next subsampled the trajectory for each timepoint group to the lowest cell number in any modality but a minimum of 100. The subsampled trajectory was then stratified into 50 bins of equal cell number or 20 bins in case of neurogenesis trajectories.

### Distribution of chromatin states during differentiation pseudotime

To assess how genomic regions change chromatin states during differentiation, we first selected regions where peaks of the three marks were detected in more than 5% of cells in any pseudotime bin. For each mark, we further determined a detection threshold by computing the median detection for all peaks in these regions in all pseudotime bins. For each modality and chromatin bin, we defined regions with peaks above this detection threshold as detected. If a region was marked by H3K27me3 and H3K4me3 in the same bin, they were annotated as bivalent, and, if they were marked by H3K27me3 and H3K27ac in the same bin, they were annotated as active promoters.

### Clustering of pseudotemporal expression patterns

To discover groups of genes with similar pseudotemporal expression patterns, we clustered smoothed expression patterns based on a dynamic time warping distance. For this, genes for clustering were selected by intersecting 6,000 highly variable genes in RNA with genes detected in more than 2% of cells in any pseudotime bin for all chromatin modalities. For these genes, the average log-normalized expression and gene activity was computed for each modality and pseudotime bin. We smoothed the mean expression for each gene’s values using a generalized additive model with a cubic spline (bs = ‘cs’), which was fit using the R package mgcv^70^. Smoothed expression over pseudotime bins for all modalities was used to compute a dynamic time warping distance between all genes using the R package dtw^73^, which was further used for *k*-means clustering with the R package FCPS^74^. For each of these clusters, we performed GO enrichment against the 6,000 most highly variable genes from RNA. One of these clusters was highly enriched for neuron-related biological processes, and the genes in this cluster were further used in subsequent analyses. Figure 4a shows a subset of the clustering that covers all pseudotemporal patterns. We provide the full list of genes in Supplementary Table 10.

### Reconstruction of the neurogenesis trajectory for the 4-month timepoint

To better understand pseudotemoral expression and chromatin modification dynamics during neurogenesis, we sought to further mitigate potential confounding factors, such as distribution of timepoints, data quality and the pseudotime inference procedure. For this, we subset the data to telencephalic NPCs and neurons from the 4-month timepoint and applied the following additional quality filters to remove outliers: H3K27ac: >1,000 peak fragments per cell; H3K4me3: >316 (10^2.5^) peak fragments per cell, <10,000 peak fragments per cell; H3K27me3: <3,162 (10^3.5^) peak fragments per cell. Pseudotime inference was subsequently performed using diffusion maps as described above. For RNA, we re-computed a set of 2,000 variable features, excluding cell cycle genes, further regressed out cell cycle scores (ScaleData()) and performed PCA (RunPCA()). We used the first 20 PCs to compute diffusion maps with the R package destiny^35^. Analogous to the CUT&Tag data, ranks along the first diffusion component were used as a pseudotemoral ordering. The trajectory was then divided into 10 or 20 bins (depending on the analysis). Supplementary Fig. 3b shows a subset of the clustering that covers the major pseudotemporal patterns. We provide the full list of genes in Supplementary Table 11.

### Detection of inflection point for neuronal genes

To examine at which point the CUT&Tag signal at neuronal genes (from clustering analysis described above) changes during neurogenesis relative to their expression, we sought to identify the first pseudotime bin in which the detection rate significantly increases for active histone modifications, chromatin accessibility and RNA expression or decreases for repressive histone modifications (Fig. 4f). For this, we used the neurogenesis pseudotime from the 4-month timepoint divided into 10 bins and defined the first bin as the baseline. For each subsequent bin, we performed differential detection analysis against the baseline using a binomial linear model as described above. Multiple testing correction was performed using the Benjamini–Hochberg method. Based on this, we determined the first bin with false discovery rate (FDR) < 0.05 and log_2_ fold change > 0.25. We performed a similar analysis in Supplementary Fig. 4i using the multiome data of the 19-gw fetal brain sample. This analysis was performed on all neuronal genes that were selected based on having maximal expression in neurons over NPCs and detection of H3K27ac in more than 5% of cells in any neuronal high-resolution cluster. From this, we computed a ‘pseudotime lag’ as the difference between the first divergent bin in the RNA and chromatin modifications.

### Analysis of multiome data of the developing human brain

We aligned the sequencing reads to the human genome and transcriptome (hg38, provided by 10x Genomics) using Cell Ranger Arc (version 2.0.0) with default parameters to obtain fragment files, peak calls and transcript counts. The data were further pre-processed and analyzed using Seurat (version 3.2)^62^ and Signac (version 1.1)^61^. Quality control was performed using the following criteria: 1,000–6,000 detected features per cell (RNA), more than 1,500 UMI counts per cell (RNA) and 1,000–50,000 detected peaks per cell (ATAC). Based on RNA expression, we further performed variable feature selection, z-scaling and PCA. The first 20 PCs were used as an input for Louvain clustering and UMAP. The clusters were manually annotated based on expression of marker genes. To specifically analyze the telencephalic neuron trajectory in these data, we extracted clusters corresponding to dorsal telencephalon NPCs, intermediate progenitors and neurons. For this subset of the data, we again performed variable feature selection, z-scaling and PCA and used the first 20 PCs to run UMAP and to compute diffusion maps with destiny^35^. Ranks along the first diffusion component were used as a pseudotemoral ordering. The velocity pseudotime was further rank transformed and divided by the total number of cells in the dataset.

### GRN analysis

To assess how the chromatin modifications shape the GRN during neurogenesis, we first inferred a GRN using Pando based on a dataset with integrated RNA and ATAC modalities over organoid development^14^. To select genomic regions for GRN inference, we performed region-to-gene linkage using the Signac function LinkPeaks() and intersected these regions with CUT&Tag peaks with more than 5% detection rate in any high-resolution cluster from all modalities. We used these regions to infer the GRN with the Pando function initiate_grn(). We further ran find_motifs() using the transcription factor motifs provided by Pando and inferred the GRN using infer_grn() with the following non-default parameters: peak_to_gene_method = ‘GREAT’, tf_cor = 0.2, downstream = 100,000 and aggregated ATAC data to high-resolution clusters called by the authors (aggregate_peaks_col = ‘highres_clusters’). We filtered this network for significant positive connections with FDR > 0.01, Pearson correlation > 0.2 and coefficient > 0. To investigate the regulation of neurogenesis using this network, we extracted a subnetwork containing all neuronal genes (from clustering analysis described above) as well as transcription factors shared by at least three of these genes. For the regulatory regions in this network, we evaluated the epigenetic status in each pseudotime bin by applying a detection threshold of 5% for each chromatin modality. Network visualization and analysis was performed with ggraph (TLP; https://ggraph.data-imaginist.com/authors.html) and tidygraph (TLP; https://tidygraph.data-imaginist.com/authors.html). The subnetwork in Fig. 4h shows genes in the neuronal cluster with priming of active epigenetic marks (a - cluster 6) at early (left) and late (right) pseudotime stages. This subnetworks includes all genes in that cluster as well as transcription factors identified in the GRN to regulate at least three of the genes within that cluster. Edges represent regulatory regions with a transcription factor binding motif, and edge color indicates epigenomic state. For example, the transcription factor binding motif for KLF7 in the vicinity of NEUROD2 is marked by H3K27me3 at the NPC stage and gains H3K27ac/H3K4me3 at the neuron stage. Instead, the two binding motifs for JUN in the vicinity of NEUROD2 remain H3K27me3 and H3K27ac marked, respectively. Node size represents pagerank centrality.

### Analysis of pseudotemporal and regional variance

To assess how expression and enrichment of genes and peaks varied during neurogenesis over pseudotime and between regions, we first selected 4,000 highly variable genes and peaks with detection in more than 5% of cells in any high-resolution cluster. Next, we computed the average expression and activity for each high-resolution cluster. We fit three Gaussian linear models for each gene *i* with mean cluster expression (Y) as the response variable and region assignment and/or pseudotime as the independent variables:

1. Y ~ region
2. Y ~ pseudotime
3. Y ~ pseudotime + region

We used the R^2^ value of these models as the fraction of variance explained by region (1), pseudotime (2) or branch and pseudotime (3).

An analogous analysis was performed to compare regional variance between astrocytes and NPCs. For each cell state, we fit a Gaussian linear model as

Y ~ region and used the R^2^ value of the model as a measure of regional variance.

### Pre-processing and integration of drug treatment scRNA-seq data

The scRNA-seq data of A395-treated organoids were pre-processed analogous to the scRNA-seq data from the developmental timecourse. We then used Harmony^75^ with default parameters to integrate the different samples. Using the Harmony integration, we performed Louvain clustering with a resolution of 0.2 and annotated the clusters based on canonical marker gene expression.

### Pre-processing of bulk CUT&Tag data

The FASTQ reads from the bulk CUT&Tag experiment were aligned to the human genome (hg38) using bwa^76^. Next, normalized bigWig files were obtained using deeptools bamCoverage^77^ with –ignoreDuplicates, -bs 200 and –normalizeUsing RPKM. To normalize bigWig files based on spike-ins, we first aligned spike-in reads to the human genome (hg38) using bwa^76^. Next, we computed scaling factors from the resulting BAM files using multiBamSummary bins with default parameters and used the result to perform normalization with bamCoverage^77^ (–ignoreDuplicates, -bs 200 and –scaleFactor). Heatmaps were generated using deeptools computeMatrix and plotHeatmap on the all H3K27me3 peaks from the stages PSC, NNE and neuroepithelium of the developmental timecourse that were detected in more than 5% of the cells.

### Differential peak analysis in the perturbation experiment

To obtain enrichment scores on the level of peaks, we summarized normalized bigWig files to the peaks from the developmental timecourse for each modality. Based on these peak intensities, we computed the log_2_ fold change of treated versus control samples for each sample and concentration separately. The log_2_ fold changes were then summarized by computing the mean for each concentration.

### Inference of terminal fate probabilities in the perturbation experiment

To better resolve the differentiation hierarchies in the perturbation data, we computed transition probabilities into terminal fates based on RNA velocity. Count matrices for the spliced and unspliced transcriptome were obtained using kallisto (version 0.46.0)^69^ by running the command line tool loompy fromfastq from the Python package loompy (version 3.0.6) (https://linnarssonlab.org/loompy/). We used scVelo (version 0.2.4)^35^ and scanpy (version 1.8.2)^70^ to perform RNA velocity analysis. The 2,000 most highly variable features were selected using the function scanpy.pp.highly_variable_genes() and used to compute moments in PCA space using the function scvelo.pp.moments() with n_neighbors = 20. RNA velocity was calculated using the function scvelo.tl.velocity() with mode = ‘stochastic’, and a velocity graph was constructed using scvelo.tl.velocity_graph() with default parameters. To order cells in the developmental trajectory, a root cell was chosen randomly from cells of the first timepoint (EB), and velocity pseudotime was computed with scvelo.tl.velocity_pseudotime(). We next computed fate probabilities into the following manually annotated terminal cell states: NNE, neural crest and neurons. For this, we used CellRank (version 1.3.0)^23^. A transition matrix was constructed with a palantir kernel (PalantirKernel()) based on velocity pseudotime. Absorption probabilities for each of the pre-defined terminal states were computed using the GPCCA estimator. Fate probabilities for each cell were visualized using a circular projection^78^. In brief, we evenly spaced terminal states around a circle and assigned each state an angle *α*_*t*_. We then computed 2D coordinates (*x*_*i*_, *y*_*i*_) from the $F\in {R}^{{Nx}{n}_{{t}}}$ transition probability matrix for N cells and ${n}_{{t}}$ terminal states as

$${x}_{i}=\sum _{t}{f}_{it}\,\cos {\alpha }_{t}$$

$${y}_{i}=\sum _{t}{f}_{it}\,\sin {\alpha }_{t}$$

To visualize enrichment of perturbed cells in this space, we used the method outlined in Nikolova et al.^79^. Here, the *k*-nearest neighbors graph (*k* = 100) was computed using Euclidean distances in fate probability space, and enrichment scores were visualized on the circular projection. Otherwise, the method was performed as described in the preprint.

### Differential gene expression analysis in the perturbation experiment

To assess the changes in gene expression upon treatment with A395, we performed DE analysis based using a logistic regression framework. To test for global DE while accounting for compositional differences, we considered the Louvain cluster label as a covariate in the model. We further accounted for sampling timepoint and sequencing depth (UMI count):

$${\rm{treatment}} \sim {\rm{Y}}\_{\rm{i}}+{\rm{n}}\_{\rm{UMI}}+{\rm{time}}\,{\rm{point}}+{\rm{louvain}}\_{\rm{cluster}}$$

We used the Seurat function FindMarkers() to perform the test for each condition separately and for all conditions combined. The resulting *P* values were FDR adjusted using the Benjamini–Hochberg method. We used a significance threshold of FDR < 0.01 and absolute log_2_ fold change > 0.1.

### Differential composition analysis

To test for compositional differences upon treatment with A395, we performed a Cochran–Mantel–Haenzel test stratified by sampling timepoint for each Louvain cluster and concentration separately. The resulting *P* value was FDR corrected, and a significance threshold of 10^−4^ was applied.

### Calculation of repression, activation and bivalency scores

To assess the epigenetic state of differentially expressed genes in different cell states, we computed repression (H3K27me3), activation (H3K4me3) or bivalency (H3K27me3 and H3K4me3) across high-resolution clusters. To compute activation and repression scores, we used the Seurat function FindModuleScore() to compute the gene activity deviation from background for H3K4me3 and H3K27me3. We computed the means of these scores for each high-resolution cluster and subsequently z-scaled them. A bivalency score was defined as zscale(H3K4me3 score) − zscale(H3K27me3 score). Here, a value close to 0 indicates bivalency, whereas positive and negative values indicate predominant activation (H3K4me3) and repression (H3K27me3), respectively.

### Transcription factor motif enrichment

To determine whether transcription factor motifs were enriched in a set of regulatory regions, we performed transcription factor motif enrichment. For this, position weight matrices (PWMs) of human transcription factor binding motifs were obtained from the CORE collection of JASPAR 2020 (ref. ^80^). Motif positions in peak regions were determined using the R package motifmatchr (version 1.14)^72^ through the Signac function FindMotifs(). We then used a Fisherʼs exact test to obtain *P* values for differential enrichment of motifs in these regions. The *P* values were FDR corrected, and a significance threshold of 0.05 was applied. We applied this analysis on region-specific peaks as well as on identified bivalent and switching peaks. Additionally, we applied this analysis to find transcription factors with putative involvement in aberrant cell fate determination upon EED inhibitor treatment. Here, we selected genomic regions in proximity (<10 kb distance) to genes with DE in ectopic clusters (FDR < 10^−4^, log_2_ fold change > 1) that were depleted upon treatment (log_2_ fold change < −1).

### Reporting summary

Further information on research design is available in the Nature Portfolio Reporting Summary linked to this article.

## Online content

Any methods, additional references, Nature Portfolio reporting summaries, source data, extended data, supplementary information, acknowledgements, peer review information; details of author contributions and competing interests; and statements of data and code availability are available at 10.1038/s41593-024-01652-0.

### Supplementary information

Supplementary Information Supplementary Figs. 1–8 and description of the supplementary tables.

Reporting Summary

**Supplementary Tables** Supplementary Tables 1–21.

## Data Availability

All raw sequencing data have been uploaded to the European Genome Phenome Archive (https://ega-archive.org/studies/EGAS50000000155). All processed data are available at https://episcape.ethz.ch, where they can be browsed interactively. Processed data can also be downloaded at 10.5281/zenodo.10471808 (ref. ^81^).

## Code availability

All code generated in the study, including analysis parameters, is available at https://github.com/quadbiolab/organoid_epigenomics and 10.5281/zenodo.10964284 (ref. ^82^).
