# Supplementary material for: Single-cell epigenomic reconstruction of developmental trajectories from pluripotency in human neural organoid systems
Source: Nat Neurosci. 2024 Jun 24;27(7):1376–86. doi: 10.1038/s41593-024-01652-0 (PMC11239525; doi:10.1038/s41593-024-01652-0)
Supplement: Supplementary file 1 — Supplementary Figs. 1–8 and description of the supplementary tables. [file 41593_2024_1652_MOESM1_ESM.pdf]

nature neuroscience

Supplementary information

https://doi.org/10.1038/s41593-024-01652-0

Single-cell epigenomic reconstruction of developmental trajectories from pluripotency in human neural organoid systems

In the format provided by the authors and unedited

Supplementary Information

Regionalization within the glial lineage

We investigated regional variance also at the chromatin level when we associate region-specific peaks of the active marks H3K27ac and H3K4me3 to the closest gene (Supplementary Fig. 2j). Interestingly, H3K27me3 at regional marker genes (LHX1, FOXG1, LHX2) showed higher gene repression variance in NPCs compared to astrocytes indicating that gene repression in astrocytes is less predictive of regional identity. Altogether, our analysis reveals that the interplay of chromatin modifications induces and stabilizes brain region diversification events.

Differential regulation of regional diversification and neuronal differentiation

We analyzed each gene across all modalities to determine how histone modifications might be differentially involved in brain region diversification versus neuronal differentiation. (Supplementary Fig. 7a-d, Supplementary Table 16, see Methods Analysis of pseudotemporal and regional variance). Overall, RNA expression and histone modifications (Supplementary Fig. 7e) showed high concordance. Variance in gene expression and H3K27ac enrichment was mostly explained by regional diversification (Supplementary Fig. 7f-g), whereas H3K27me3 showed similar variance across regional branches and over pseudotime. H3K4me3 showed lower variance across regional branches than over pseudotime (Supplementary Fig. 7g). We characterized the genome-wide distribution of branch-specific peaks. This revealed that the majority (~75%) of H3K4me3 peaks was located at promoter regions, while for H3K27ac and H3K27me3, a lower percentage (60%) of branch-specific peaks was found at promoter regions and instead, 40% of these peaks were found in distal regions (Supplementary Fig. 7h).

a

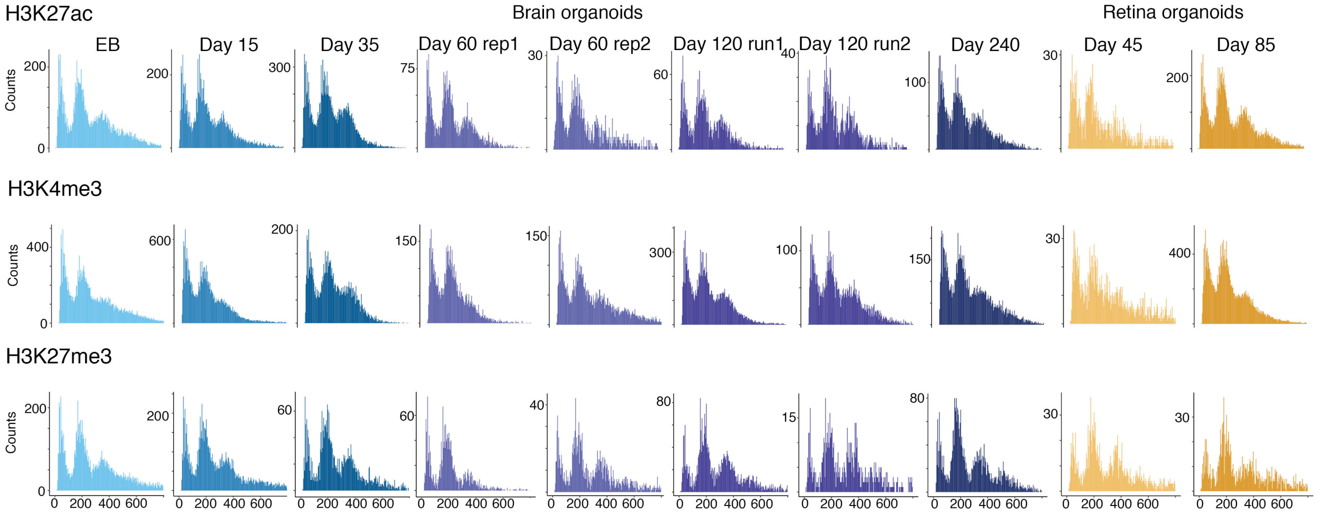

b

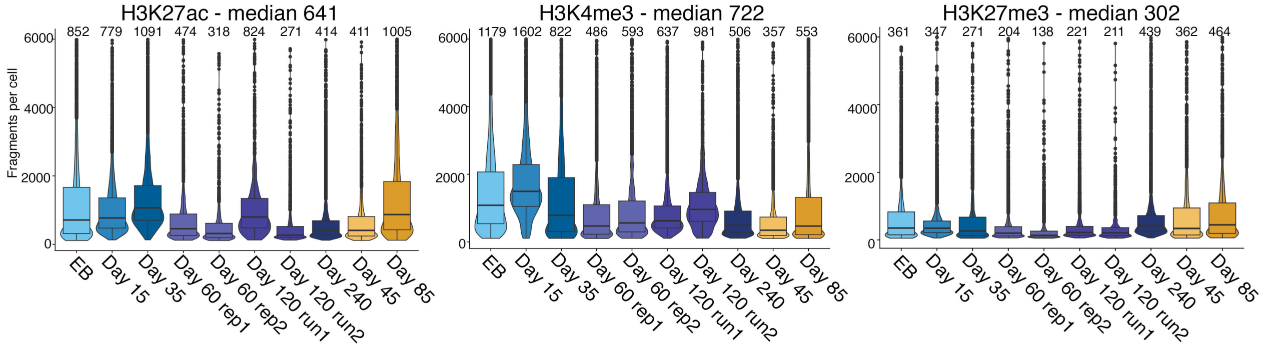

c

Citation	Method	Tissue/Cell lines	Median fragments/cell
Zenk & Fleck et al. 2023<br>(presented study)	scCUT&Tag	Human brain and retina organoids	H3K27ac - 641 (range: 271 - 1005)<br>H3K4me3 - 722 (range: 357 - 1602)<br>H3K27me3 - 302 (range: 138 - 464)
Zhang et al. 2022<br>Nat. Biotech.	scCUT&Tag pro	Human peripheral blood mononuclear cells (PBMC)	H3K27ac - 199<br>H3K4me3 - 316<br>H3K27me3 - 501
Bartosovic et al.<br>2021 Nat. Biotech.	scCUT&Tag	Postnatal mouse brain (FACS sorted) and<br>mix of mouse embryonic stem cells (mESC, C57Bl/6J origin),<br>mouse embryonic fibroblasts (NIH-3T3) (ATCC),<br>mouse oligodendrocyte progenitor model cell line (Oli-neu)	H3K27ac - 453<br>H3K4me3 - ~420<br>H3K27me3 - 597
Gopalan et al.<br>2021 Mol. Cell.	multi-CUT&Tag	E14 mouse embryonic stem cell line<br>5-4 mouse trophoblast stem cell line	not reported, cut-off of 200 cut sites per cell used
Wu et al. 2021<br>Nat. Biotech.	scCUT&Tag	Human peripheral blood mononuclear cells (PBMC)	H3K27me3 - 1110 (range: 954 - 1230)<br>H3K27ac - 1047

d

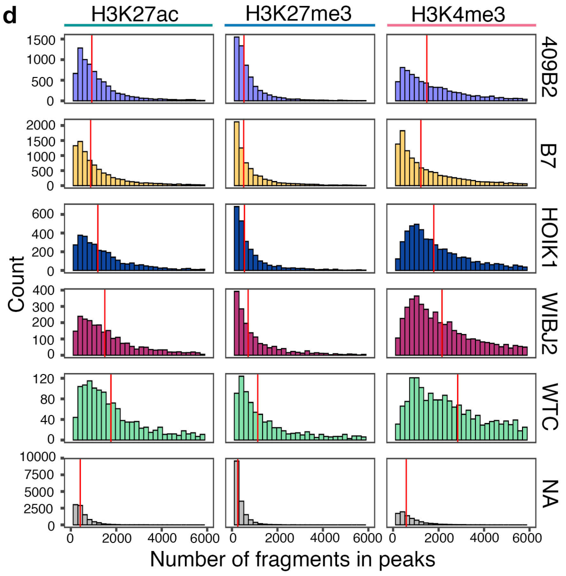

Supplementary Fig. 1 Quality metrics of the scCUT&Tag dataset

a, Histograms showing fragment length distribution for all samples (different timepoints including replicates) and modalities (H3K27ac, H3K4me3, H3K27me3), showing a clear nucleosome size pattern b, Violin plots showing the distribution of detected fragments per cell for each time point and chromatin modality (H3K27ac: n=33533, H3K27me3: n=34357, H3K2me3: n=42057, 8 independent timepoints each). The median value of each boxplot is indicated at the top (median +/- Q1/Q3). c, Table comparing median read per cell as a measure of data quality between different scCUT&Tag publications and the presented study (Zenk & Fleck et al. 2023). d, Histograms comparing the distribution of peak fragments per cell for each histone modification in all cell lines in the presented dataset. The red line demarcates the median.

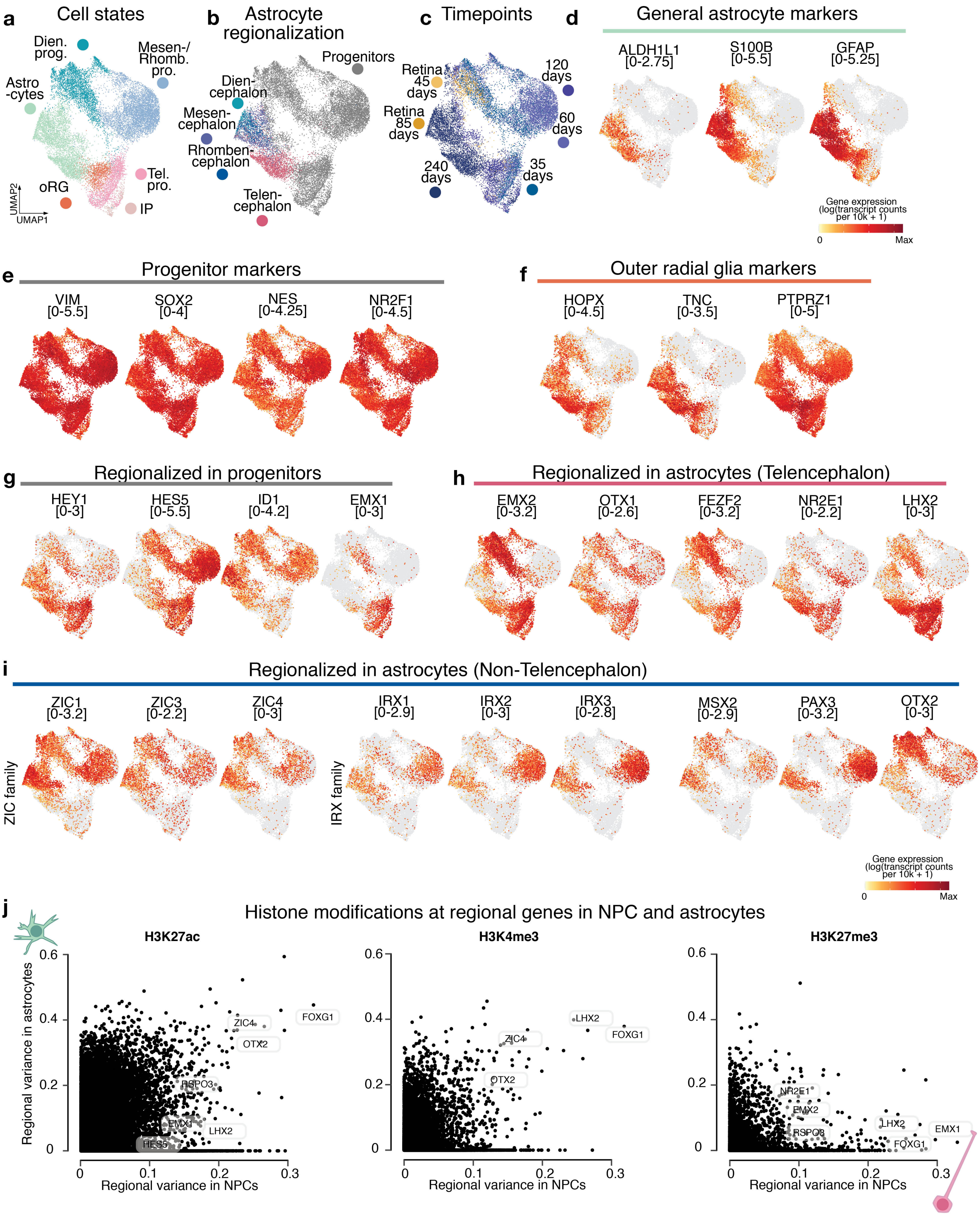

a Cell states
Dien. prog.
Mesen-/ Rhomb. pro.
Astro -cytes
oRG
Tel. pro.
IP
UMAP2
UMAP1
b Astrocyte regionalization
Progenitors
Dien- cephalon
Mesen- cephalon
Rhomben- cephalon
Telen- cephalon
c Timepoints
Retina 45 days
Retina 85 days
120 days
60 days
35 days
240 days
d General astrocyte markers
ALDH1L1 [0-2.75]
S100B [0-5.5]
GFAP [0-5.25]
Gene expression (log(transcript counts per 10k + 1)
0
Max
e Progenitor markers
VIM [0-5.5]
SOX2 [0-4]
NES [0-4.25]
NR2F1 [0-4.5]
f Outer radial glia markers
HOPX [0-4.5]
TNC [0-3.5]
PTPRZ1 [0-5]
g Regionalized in progenitors
HEY1 [0-3]
HES5 [0-5.5]
ID1 [0-4.2]
EMX1 [0-3]
h Regionalized in astrocytes (Telencephalon)
EMX2 [0-3.2]
OTX1 [0-2.6]
FEZF2 [0-3.2]
NR2E1 [0-2.2]
LHX2 [0-3]
i Regionalized in astrocytes (Non-Telencephalon)
ZIC family
ZIC1 [0-3.2]
ZIC3 [0-2.2]
ZIC4 [0-3]
IRX family
IRX1 [0-2.9]
IRX2 [0-3]
IRX3 [0-2.8]
MSX2 [0-2.9]
PAX3 [0-3.2]
OTX2 [0-3]
Gene expression (log(transcript counts per 10k + 1)
0
Max
j Histone modifications at regional genes in NPC and astrocytes
H3K27ac
H3K4me3
H3K27me3
Regional variance in astrocytes
Regional variance in NPCs
FOXG1
ZIC4
OTX2
RSPO3
EMX1
LHX2
HES5
NR2E1
EMX2

Supplementary Fig. 2 Astrocyte regionalization signatures in human brain organoids

a-c, UMAP embedding of NPCs and astrocytes from the 30-240 day time points colored by cell state (a), brain region (b), and time point (c). d, UMAP embedding colored by gene expression (log(transcript counts per 10k + 1)) of general astrocyte marker genes. e, UMAP embedding colored by gene expression of general progenitor marker genes. f, UMAP embedding colored by gene expression of general outer radial glia marker genes. g-i, UMAP embedding colored by expression of genes that show a brain region specific expression pattern in NPCs (e), are specifically expressed in forebrain astrocytes and NPCs (f) are specifically expressed in non-forebrain astrocytes and NPCs (g). j, Scatter plot comparing the regional variance (R^2) of histone modification enrichment (for H3K27ac, H3K4me3, H3K27me3) between astrocytes and NPCs. This suggests that gene repression (H3K27me3) is less predictive of regional identity in astrocytes compared to NPCs (see Methods Analysis of pseudotemporal and regional variance for details).

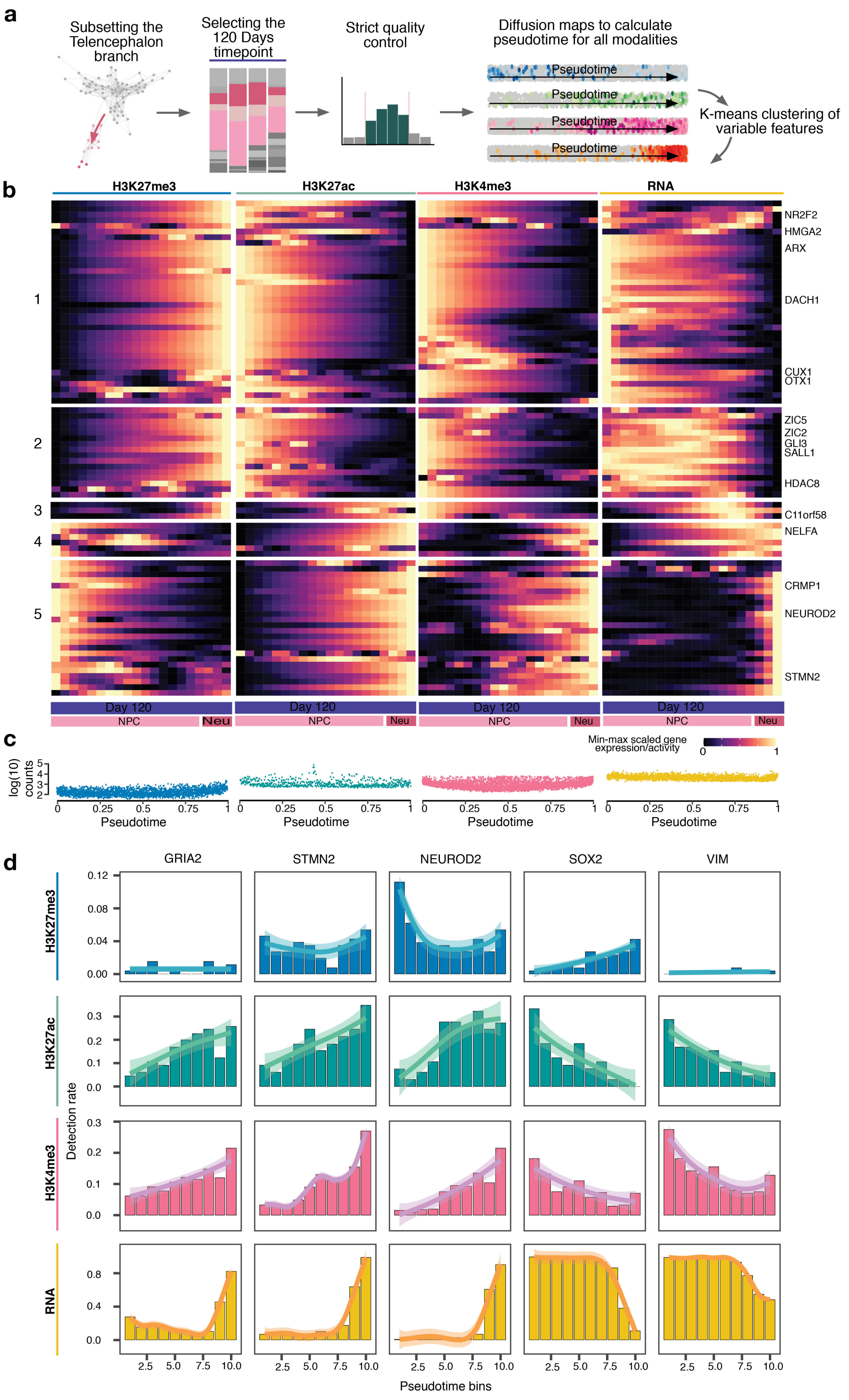

a
Subsetting the Telencephalon branch
Selecting the 120 Days timepoint
Strict quality control
Diffusion maps to calculate pseudotime for all modalities
Pseudotime
K-means clustering of variable features
b
H3K27me3
H3K27ac
H3K4me3
RNA
1
2
3
4
5
NR2F2
HMGA2
ARX
DACH1
CUX1
OTX1
ZIC5
ZIC2
GLI3
SALL1
HDAC8
C11orf58
NELFA
CRMP1
NEUROD2
STMN2
Day 120
NPC
Neu
c
Min-max scaled gene expression/activity
log(10) counts
Pseudotime
d
GRIA2
STMN2
NEUROD2
SOX2
VIM
Detection rate
Pseudotime bins

Supplementary Fig. 3 Pseudotemporal reconstruction of dorsal telencephalic neuron differentiation using diffusion maps

a, Schematic of the analysis. We subset the telencephalon branch and isolated only cells of the 120 days time point. The cells were again filtered for quality and pseudotime for all modalities was calculated using diffusion maps (see Methods Reconstruction of the neurogenesis trajectory for the 4 months time point for details). b, Heatmap showing scaled and smoothed gene activity scores (log(fragment counts per 10k + 1) on gene body +2kb promoter region) for H3K27me3, H3K27ac and H3K4me3 as well as RNA expression (log(transcript counts per 10k + 1)) over the telencephalic neuron differentiation trajectory from pluripotency. Pseudotime was binned and genes were K-means clustered based on average expression/activity of all marks and RNA in all bins (Supplementary Table 11 contains all clusters). Using this strictly refined analysis we could again identify a neuronal cluster that clearly showed priming with active chromatin modifications. c, Jitter plot showing even UMI count and fragment counts versus pseudotime for all modalities. d, Barplot showing fragment detection rate in pseudotime bins at selected example genes for all modalities. The line indicates smoothing with generalized additive models.

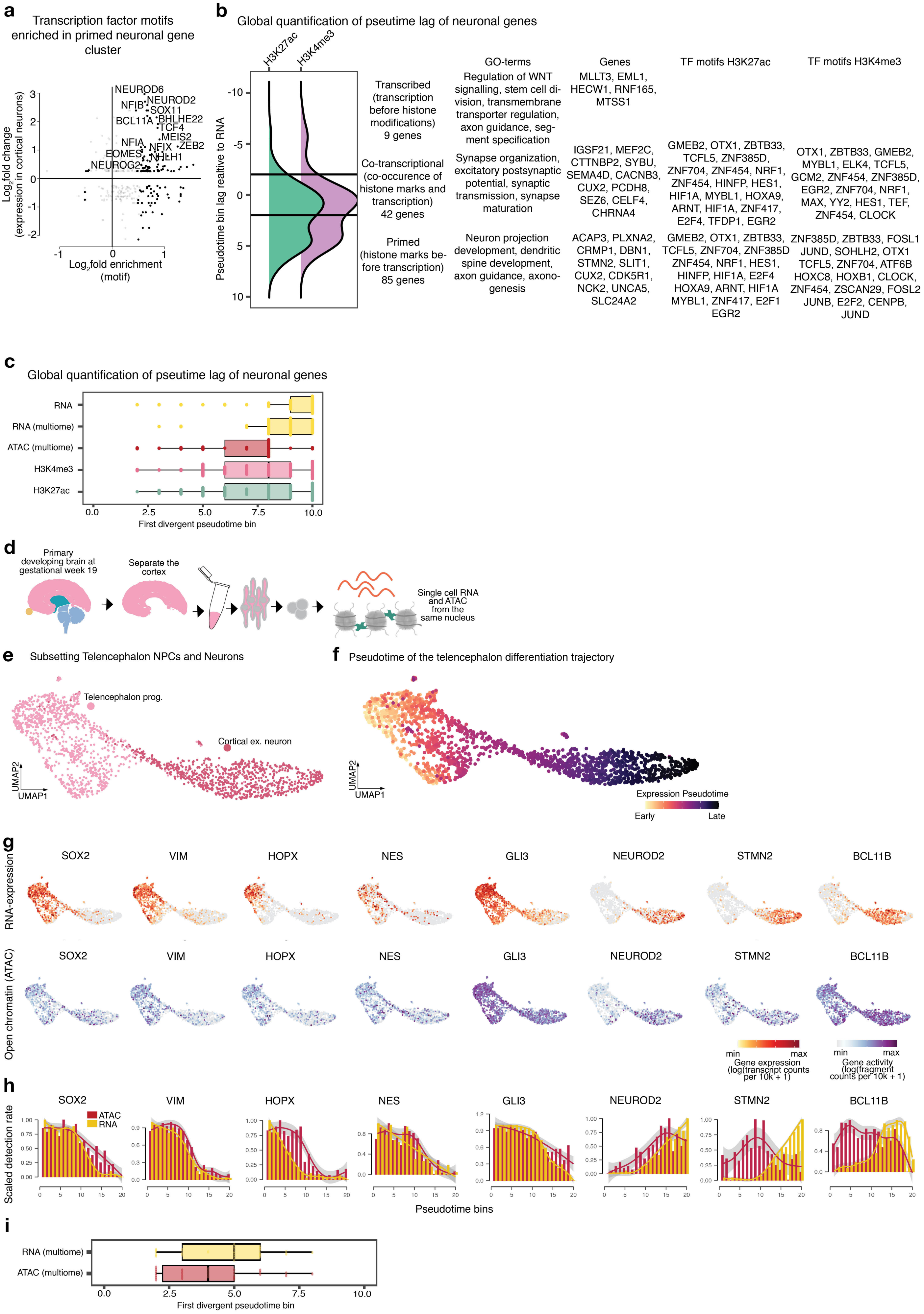
a
Transcription factor motifs enriched in primed neuronal gene cluster
NEUROD6
NFIB
NEUROD2
SOX11
BCL11A
BHLHE22
TCF4
MEIS2
NFIA
NFIX
ZEB2
EOMES
NHLH1
NEUROG2
Log2fold change (expression in cortical neurons)
Log2fold enrichment (motif)
b
Global quantification of pseutime lag of neuronal genes
H3K27ac
H3K4me3
Pseudotime bin lag realtive to RNA
GO-terms
Genes
TF motifs H3K27ac
TF motifs H3K4me3
Transcribed (transcription before histone modifications) 9 genes
Regulation of WNT signalling, stem cell division, transmembrane transporter regulation, axon guidance, segment specification
MLLT3, EML1, HECW1, RNF165, MTSS1
Co-transcriptional (co-occurence of histone marks and transcription) 42 genes
Synapse organization, excitatory postsynaptic potential, synaptic transmission, synapse maturation
IGSF21, MEF2C, CTTNBP2, SYBU, SEMA4D, CACNB3, CUX2, PCDH8, SEZ6, CELF4, CHRNA4
GMEB2, OTX1, ZBTB33, TCFL5, ZNF385D, ZNF704, ZNF454, NRF1, ZNF454, HINFP, HES1, HIF1A, MYBL1, HOXA9, ARNT, HIF1A, ZNF417, E2F4, TFDP1, EGR2
OTX1, ZBTB33, GMEB2, MYBL1, ELK4, TCFL5, GCM2, ZNF454, ZNF385D, EGR2, ZNF704, NRF1, MAX, YY2, HES1, TEF, ZNF454, CLOCK
Primed (histone marks before transcription) 85 genes
Neuron projection development, dendritic spine development, axon guidance, axonogenesis
ACAP3, PLXNA2, CRMP1, DBN1, STMN2, SLIT1, CUX2, CDK5R1, NCK2, UNCA5, SLC24A2
GMEB2, OTX1, ZBTB33, TCFL5, ZNF704, ZNF385D ZNF454, NRF1, HES1, HINFP, HIF1A, E2F4 HOXA9, ARNT, HIF1A MYBL1, ZNF417, E2F1 EGR2
ZNF385D, ZBTB33, FOSL1 JUND, SOHLH2, OTX1 TCFL5, ZNF704, ATF6B HOXC8, HOXB1, CLOCK, ZNF454, ZSCAN29, FOSL2 JUNB, E2F2, CENPB, JUND
c
Global quantification of pseutime lag of neuronal genes
RNA
RNA (multiome)
ATAC (multiome)
H3K4me3
H3K27ac
First divergent pseudotime bin
d
Primary developing brain at gestational week 19
Separate the cortex
Single cell RNA and ATAC from the same nucleus
e
Subsetting Telencephalon NPCs and Neurons
Telencephalon prog.
Cortical ex. neuron
UMAP2
UMAP1
f
Pseudotime of the telencephalon differentiation trajectory
Expression Pseudotime
Early
Late
g
RNA-expression
Open chromatin (ATAC)
SOX2
VIM
HOPX
NES
GLI3
NEUROD2
STMN2
BCL11B
min
max
Gene expression (log(transcript counts per 10k + 1)
Gene activity (log(fragment counts per 10k + 1)
h
ATAC
RNA
Scaled detection rate
Pseudotime bins
i
RNA (multiome)
ATAC (multiome)
First divergent pseudotime bin

Supplementary Fig. 4 Global analysis of neuronal genes in organoids and pseudotime reconstruction of neurogenesis in the primary developing cortex reveal widespread priming.

a, Transcription factor motif enrichment in peaks on primed neuronal genes (gene body +2kb promoter region, see Methods Transcription factor motif enrichment)(cluster 6, Fig. 4a) plotted against their expression fold change in cortical neurons. Colored dots indicate a significant motif enrichment by Fisher exact test FDR<0.01. b, Ridge plots showing the distribution of pseudotime lag between active histone modifications (H3K27ac and H3K4me3) and RNA expression. Positive values indicate histone modifications preceding RNA, negative values the opposite. (see Methods Detection of inflection point for neuronal genes). Neuronal genes were selected based on having maximal expression in neurons over NPCs and detection of H3K27ac in more than 5% of cells in any neuronal high-resolution cluster. Primed genes show an enrichment of GO-terms related to neuron projection and axonogenesis, while co-transcriptionally marked genes are enriched for terms related to synapse organization. c, Boxplot (median +/- Q1/Q3) showing the shift in pseudotime between the establishment of active histone modifications, chromatin accessibility and RNA expression on the same set of neuronal genes as b (n=357 genes). d, Schematic of the multiome experiment in the primary developing brain. e, UMAP embedding of the cortical neuron trajectory from the gw 19 primary developing brain multiome data colored by cell state. f, UMAP embedding colored by pseudotime. g, UMAP embedding as is (b) colored by expression (log(normalized transcript counts +1)) of different neuronal and progenitor marker genes (top) and gene activity based on chromatin accessibility (log(fragment counts per 10k + 1) on gene body +2kb promoter region) (bottom). h, Barplots with smoothed lines showing detection rate of scATAC fragments and scRNA expression over pseudotime measured from the same cells of a primary human developing embryonic brain at gw 19. Neuronal genes (NEUROD2, STMN2, BCL11B) show priming of chromatin. i, Boxplot (median +/- Q1/Q3) showing the shift in pseudotime between chromatin accessibility and RNA expression in the fetal cortex on the same set of neuronal genes as in b (n=357

genes). More details on the analysis can be found in the method section on Detection of inflection point for neuronal genes.

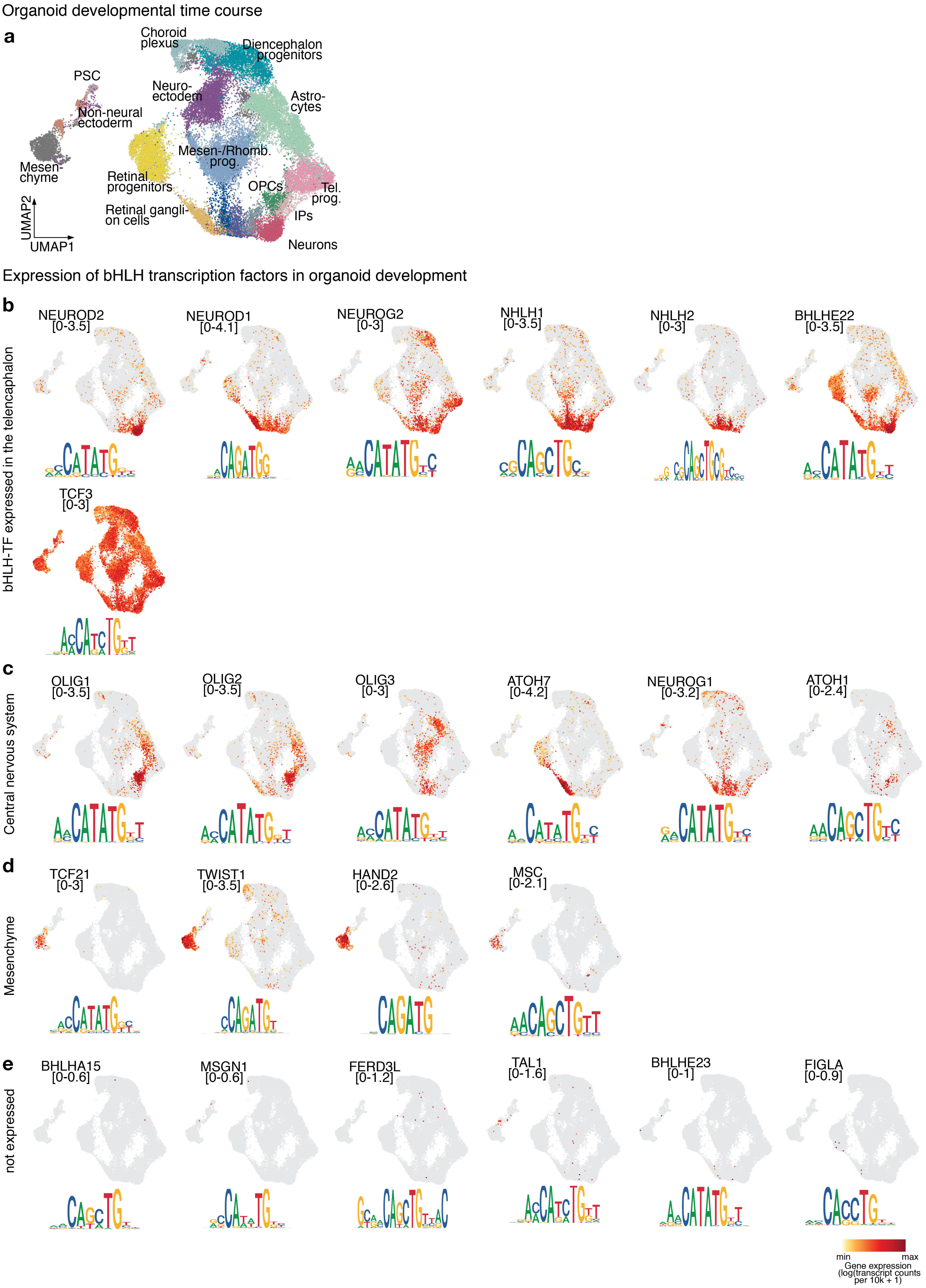

Organoid developmental time course
a
Choroid plexus
Diencephalon progenitors
PSC
Neuro-ectoderm
Astro-cytes
Non-neural ectoderm
Mesen-/Rhomb. prog.
Mesen-chyme
Retinal progenitors
OPCs
Tel. prog.
IPs
Retinal gangli-on cells
Neurons
UMAP2
UMAP1
Expression of bHLH transcription factors in organoid development
b
bHLH-TF expressed in the telencephalon
NEUROD2 [0-3.5]
NEUROD1 [0-4.1]
NEUROG2 [0-3]
NHLH1 [0-3.5]
NHLH2 [0-3]
BHLHE22 [0-3.5]
TCF3 [0-3]
c
Central nervous system
OLIG1 [0-3.5]
OLIG2 [0-3.5]
OLIG3 [0-3]
ATOH7 [0-4.2]
NEUROG1 [0-3.2]
ATOH1 [0-2.4]
d
Mesenchyme
TCF21 [0-3]
TWIST1 [0-3.5]
HAND2 [0-2.6]
MSC [0-2.1]
e
not expressed
BHLHA15 [0-0.6]
MSGN1 [0-0.6]
FERD3L [0-1.2]
TAL1 [0-1.6]
BHLHE23 [0-1]
FIGLA [0-0.9]
min
max
Gene expression (log(transcript counts per 10k + 1)

Supplementary Fig. 5 Expression analysis of bHLH transcription factors in the time course

a, UMAP embedding of scRNA-seq data with cells colored and labeled by cell state (IP - intermediate progenitor, PSC - pluripotent stem cell, RGCs - retinal ganglion cells, RPs - retinal progenitors). b, UMAP embedding of the developmental time course as in (a) colored by expression of bHLH transcription factors (log(transcript counts per 10k + 1)) with expression in the telencephalon branch and their corresponding motif. In particular NEUROG2 and BHLHE22 show very similar motifs and expression profile compared to NEUROD2 and might also act in the GRN (Fig. 4h) c, bHLH transcription factors with expression in the central nervous system. d, bHLH transcription factors with expression in the mesenchyme. e, bHLH transcription factors not expressed in the time course.

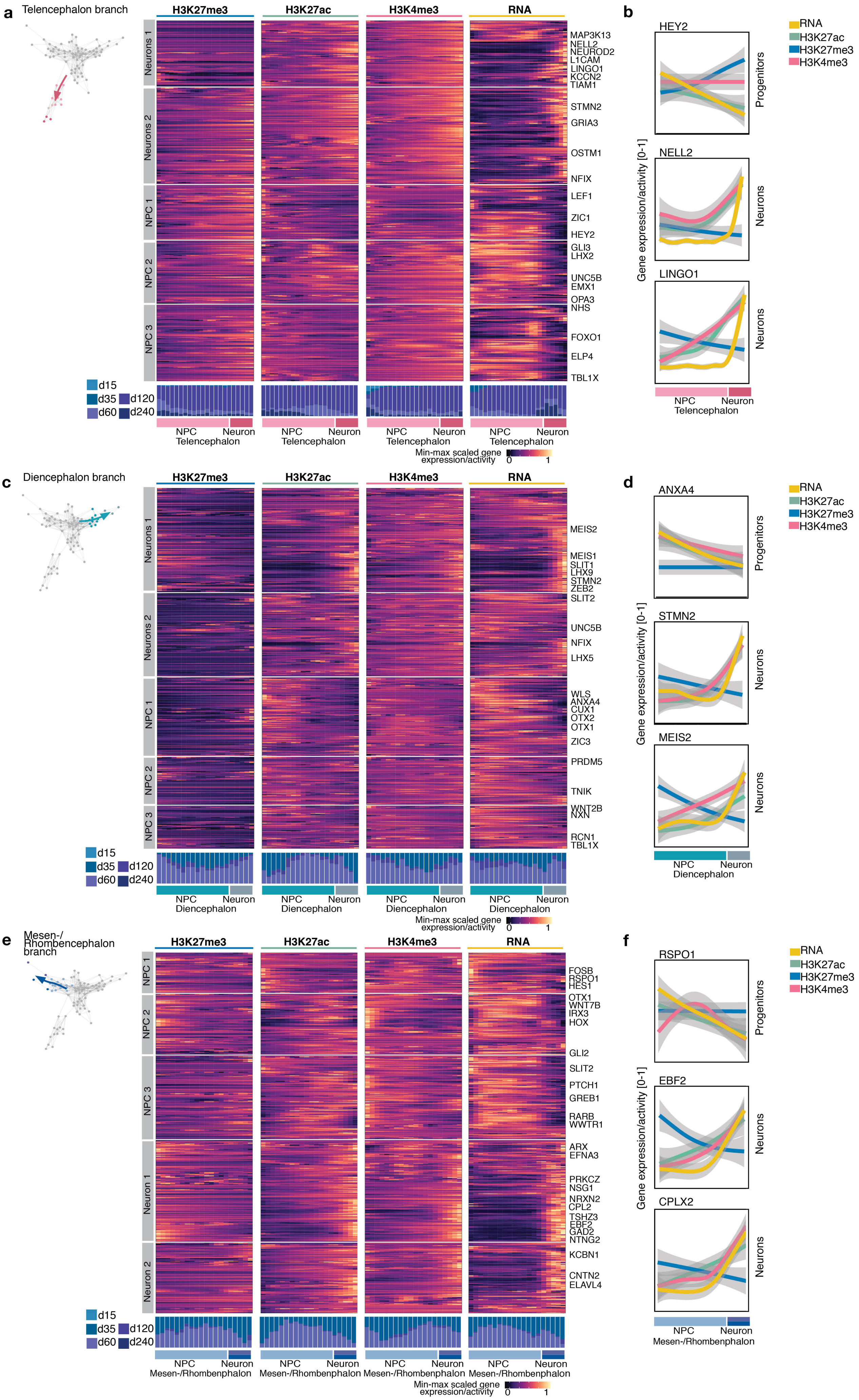
a
Telencephalon branch
H3K27me3
H3K27ac
H3K4me3
RNA
Neurons 1
Neurons 2
NPC 1
NPC 2
NPC 3
MAP3K13
NELL2
NEUROD2
L1CAM
LINGO1
KCCN2
TIAM1
STMN2
GRIA3
OSTM1
NFIX
LEF1
ZIC1
HEY2
GLI3
LHX2
UNC5B
EMX1
OPA3
NHS
FOXO1
ELP4
TBL1X
d15
d35
d120
d60
d240
NPC
Neuron
Telencephalon
Min-max scaled gene expression/activity
0
1
b
HEY2
NELL2
LINGO1
Progenitors
Neurons
Gene expression/activity [0-1]
RNA
H3K27ac
H3K27me3
H3K4me3
c
Diencephalon branch
MEIS2
MEIS1
SLIT1
LHX9
STMN2
ZEB2
SLIT2
UNC5B
NFIX
LHX5
WLS
ANXA4
CUX1
OTX2
OTX1
ZIC3
PRDM5
TNIK
WNT2B
NXN
RCN1
TBL1X
Diencephalon
d
ANXA4
STMN2
MEIS2
e
Mesen-/Rhombencephalon branch
NPC 1
NPC 2
NPC 3
Neuron 1
Neuron 2
FOSB
RSPO1
HES1
OTX1
WNT7B
IRX3
HOX
GLI2
SLIT2
PTCH1
GREB1
RARB
WWTR1
ARX
EFNA3
PRKCZ
NSG1
NRXN2
CPL2
TSHZ3
EBF2
GAD2
NTNG2
KCBN1
CNTN2
ELAVL4
Mesen-/Rhombenphalon
f
RSPO1
EBF2
CPLX2

Supplementary Fig. 6 Pseudotime reconstruction of dynamic changes of all modalities during neurogenesis in different developing organoid brain regions

a, Heatmap showing scaled gene activity scores for H3K27me3, H3K27ac and H3K4me3 as well as RNA expression over the telencephalic neuron differentiation trajectory from NPCs. Pseudotime was binned and genes were K-means clustered based on average expression/activity of all marks and RNA in all bins. (The averaged pseudotemporal gene expression and activities of cluster 1 are shown as line plots in Fig. 4j, as well as their GO term enrichment) b, Line plots showing generalized additive model-based smoothing of pseudotemporal gene expression and gene activities of selected examples from multiple K-means clusters. HEY2 is expressed at the progenitor state, showing alignment of pseudotimes. NELL2 and LINGO1 are expressed in neurons and exhibit epigenetic priming with activating marks. c, Same as (a) for the diencephalic neuron differentiation trajectory from NPCs. (The averaged pseudotemporal gene expression and activities of cluster 1 are shown as line plots in Fig. 4j, as well as their GO term enrichment) d, Line plots showing smoothed pseudotemporal gene expression and gene activities of selected examples from multiple K-means clusters. ANXA4 is expressed at the progenitor state, showing alignment of pseudotimes. STMN2 and MEIS2 are expressed in neurons and exhibit epigenetic priming with activating marks. e, Same as (a) for the rhomben- and mesencephalic neuron differentiation trajectory from NPCs. (The averaged pseudotemporal gene expression and activities of cluster 4 are shown as line plots in Fig. 4j, as well as their GO term enrichment) f, Line plots showing smoothed pseudotemporal gene expression and gene activities of selected examples from multiple K-means clusters. RSPO1 is expressed at the progenitor state, showing alignment of pseudotimes. EBF2 and CPLX2 are expressed in neurons and exhibit epigenetic priming with activating marks.

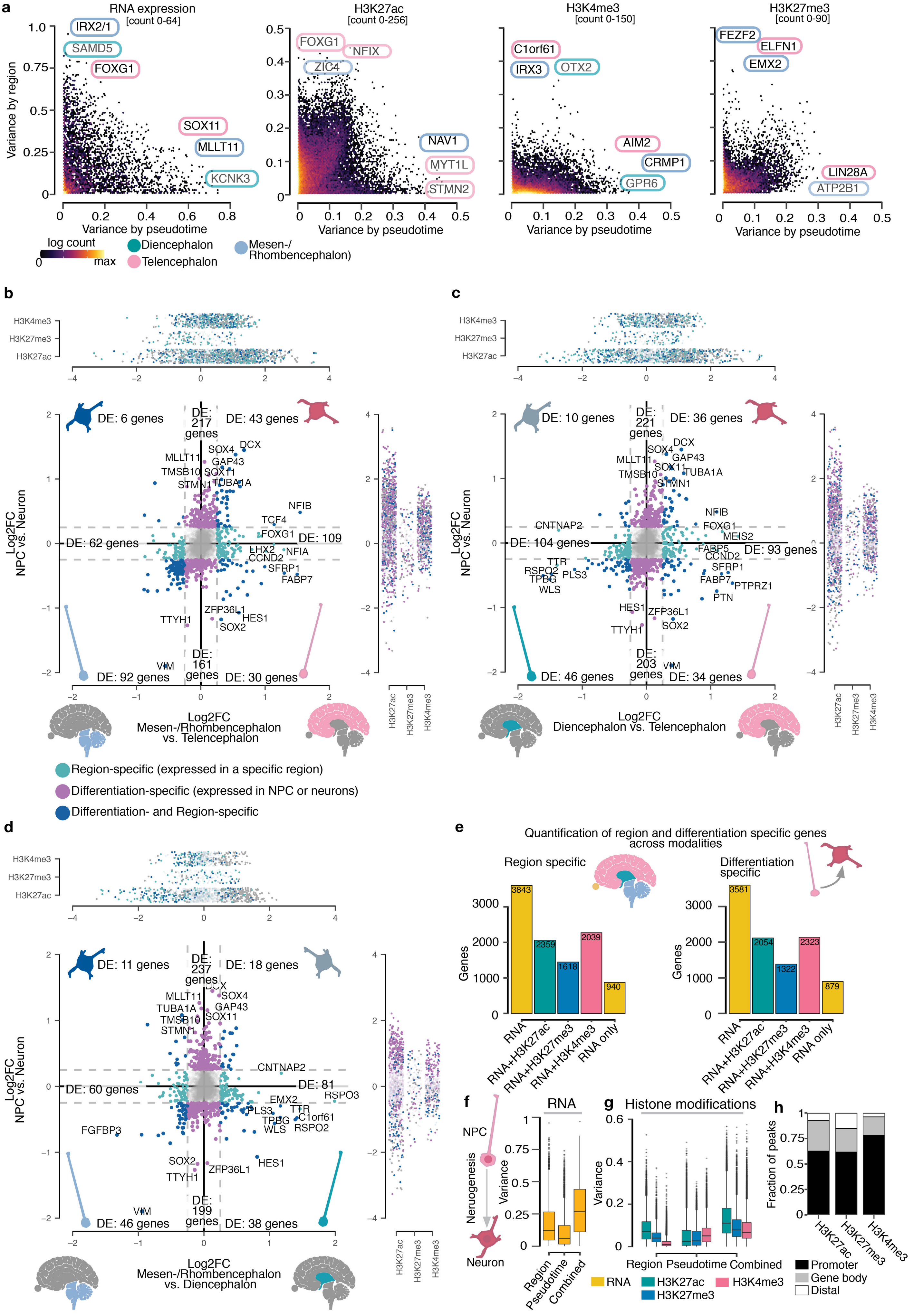
a
RNA expression
[count 0-64]
H3K27ac
[count 0-256]
H3K4me3
[count 0-150]
H3K27me3
[count 0-90]
Variance by region
Variance by pseudotime
IRX2/1
SAMD5
FOXG1
SOX11
MLLT11
KCNK3
NFIX
ZIC4
NAV1
MYT1L
STMN2
C1orf61
IRX3
OTX2
AIM2
CRMP1
GPR6
FEZF2
ELFN1
EMX2
LIN28A
ATP2B1
log count
0
max
Diencephalon
Telencephalon
Mesen-/
Rhombencephalon)
b
c
d
H3K4me3
H3K27me3
H3K27ac
DE: 6 genes
DE: 217 genes
DE: 43 genes
DE: 62 genes
DE: 109
DE: 92 genes
DE: 161 genes
DE: 30 genes
Log2FC
NPC vs. Neuron
Log2FC
Mesen-/Rhombencephalon
vs. Telencephalon
DE: 10 genes
DE: 221 genes
DE: 36 genes
DE: 104 genes
DE: 93 genes
DE: 46 genes
DE: 203 genes
DE: 34 genes
Log2FC
Diencephalon vs. Telencephalon
Region-specific (expressed in a specific region)
Differentiation-specific (expressed in NPC or neurons)
Differentiation- and Region-specific
DE: 11 genes
DE: 237 genes
DE: 18 genes
DE: 60 genes
DE: 81
DE: 46 genes
DE: 199 genes
DE: 38 genes
Log2FC
Mesen-/Rhombencephalon
vs. Diencephalon
e
Quantification of region and differentiation specific genes
across modalities
Region specific
Differentiation
specific
Genes
3843
2359
1618
2039
940
3581
2054
1322
2323
879
RNA
RNA+H3K27ac
RNA+H3K27me3
RNA+H3K4me3
RNA only
f
RNA
NPC
Neruogenesis
Neuron
Variance
Region
Pseudotime
Combined
g
Histone modifications
Region Pseudotime Combined
RNA
H3K27ac
H3K4me3
H3K27me3
h
Fraction of peaks
H3K27ac
H3K27me3
H3K4me3
Promoter
Gene body
Distal

Supplementary Fig. 7 Comparing differentiation- and region-specific gene expression between different brain regional branches

a, Scatter plot showing explained variance over pseudotime (x-axis) and regional branches (y-axis) for gene expression for RNA (top) and gene activity for all histone modifications (see Supplementary Table 16 for details). b, Scatter plot showing the log2 fold change of gene expression between neurons and progenitors (y-axis) and telencephalon and mesen/rhombencephalon (x-axis). The scatter-plots on the right and top show the same gene set comparing the log2 fold change of the individual histone modifications. c, Scatter plot showing the log2 fold change of gene expression between neurons and progenitors (y-axis) and diencephalon and telencephalon (x-axis). The scatter-plots on the right and top show the same gene set comparing the log2 fold change of the individual histone modifications. d, Scatter plot showing the log2 fold change of gene expression between neurons and progenitors (y-axis) and mesen/rhombencephalon and diencephalon (x-axis). The scatter-plots on the right and top show the same gene set comparing the log2 fold change of the individual histone modifications. Using this representation genes that are involved in differentiation (change over pseudotime) and regionalization (change between different brain regions) can be identified. e, Barplot showing the overlap for region and differentiation specific genes detected with each modality (RNA and histone modifications). f, Boxplot (median +/- Q1/Q3) showing gene expression variance over regional branches, pseudotime and both measures combined (n=20641 genes). g, Boxplot (median +/- Q1/Q3) showing gene activity (log(fragment counts per 10k + 1) on gene body +2kb promoter region) variance over regional branches, pseudotime and combined (n=20641 genes). h, Barplot showing the genome-wide distribution of branch-specific peaks for each histone modification. (see Methods Analysis of pseudotemporal and regional variance for details)

Uncropped Western Blot signal shown in Extended Data Fig. 9b

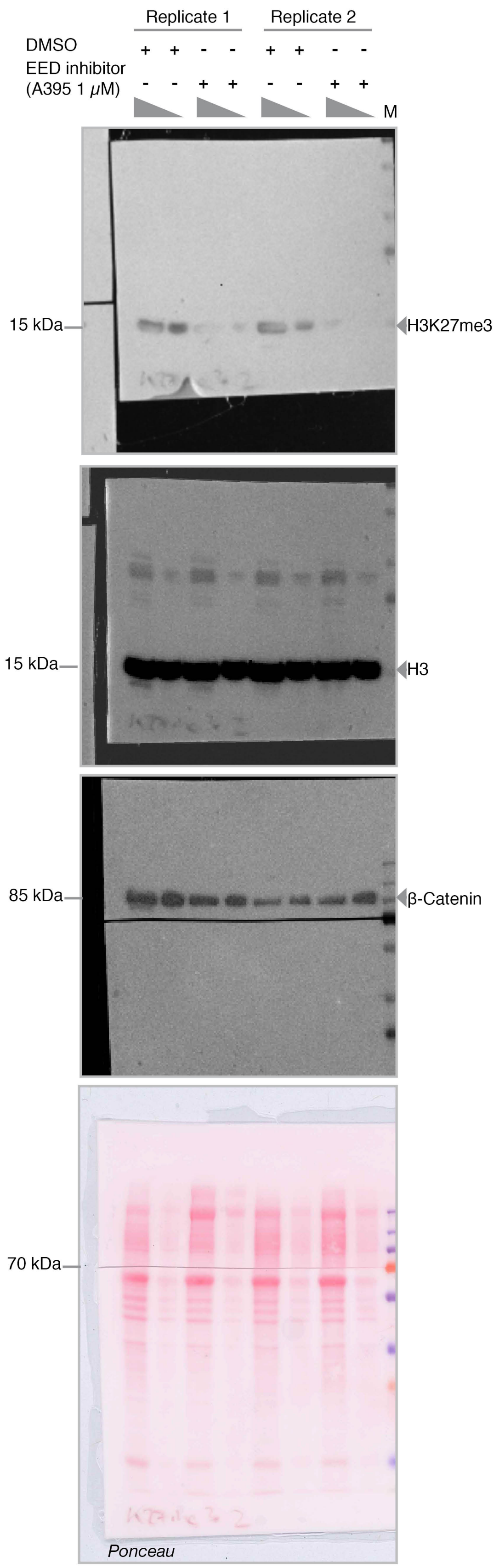

Supplementary Fig. 8 Uncropped Western Blot

Western Blot of cellular extracts of 15 day old organoids shows depletion of H3K27me3 upon treatment with 1 μM A395. H3, β-Catenin and Ponceau serve as loading controls.

Supplementary Tables:

Supp_Data_1_all_branches_peaks.csv, contains the top 50 peaks of H3K27me3, H3K4me3 and H3K27ac from all regional branches and cell states for the different cell lines. P-values were derived from a likelihood ratio test and were multiple testing corrected using the Benjamini-Hochberg method.

Supp_Data_2_lineage_peaks_GREAT_enrich.tsv, contains GO terms shown in Extended Data Fig. 5a-c. P-values were derived from a hypergeometric test and were multiple testing corrected using the Benjamini-Hochberg method.

Supp_Data_3_TF_motifs_regional_branches.tsv, contains enriched transcriptions factor motifs shown in Extended Data Fig. 5d-f. P-values were derived from a two-sided fisher exact test and were multiple testing corrected using the Benjamini-Hochberg method.

Supp_Data_4_H3K4me3_bivalent.tsv, contains filtered regions bivalent for H3K27me3 and H3K4me3 between the neuroepithelium and the regional branches (Extended Data Fig. 6a-c). P-values were derived from a likelihood ratio test and were multiple testing corrected using the Benjamini-Hochberg method.

Supp_Data_5_TF_motifs_bivalent.tsv, contains enriched transcriptions factor motifs shown in Extended Data Fig. 6d. P-values were derived from a two-sided fisher exact test and were multiple testing corrected using the Benjamini-Hochberg method.

Supp_Data_6_H3K27ac_switches.tsv, contains filtered regions switching between H3K27me3 and H3K27ac in the regional branches (Fig. 2e). P-values were derived from a likelihood ratio test and were multiple testing corrected using the Benjamini-Hochberg method.

Supp_Data_7_TF_motifs_switches.tsv, contains enriched transcriptions factor motifs shown in Extended Data Fig. 6e. P-values were derived from a likelihood ratio test and were multiple testing corrected using the Benjamini-Hochberg method.

Supp_Data_8_all_peaks_GREAT_enrich.tsv, contains GREAT annotation for the clusters shown in Extended Data Fig. 7f. P-values were derived from a hypergeometric test and were multiple testing corrected using the Benjamini-Hochberg method.

Supp_Data_9_all_peaks_TF_motifs.tsv contains enriched transcriptions factor motifs for the clusters shown in Extended Data Fig. 7f. P-values were derived from a two-sided fisher exact test and were multiple testing corrected using the Benjamini-Hochberg method.

Supp_Data_10_ctx_trajectory_genes.tsv, all genes and cluster annotation of Fig. 4a.

Supp_Data_11_ctx_trajector_4m_genes.tsv, all genes and cluster annotation Supplementary Fig. 3b.

Supp_Data_12_ctx_primed_GRN.tsv, regions and enriched histone modifications underlying Fig. 4h. P-values were derived from a likelihood ratio test and were multiple testing corrected using the Benjamini-Hochberg method.

Supp_Data_13_ctx_pt_GO.tsv, contains the clusters and GO analysis of Fig. 4j and Supplementary Fig. 6a. P-values were derived from a hypergeometric test and were multiple testing corrected using the Benjamini-Hochberg method.

Supp_Data_14_dien_pt_GO.tsv, contains the clusters and GO analysis of Fig. 4j and Supplementary Fig. 6c. P-values were derived from a hypergeometric test and were multiple testing corrected using the Benjamini-Hochberg method.

Supp_Data_15_mrh_pt_GO.tsv, contains the clusters and GO analysis of Fig. 4j and Supplementary Fig. 6e. P-values were derived from a hypergeometric test and were multiple testing corrected using the Benjamini-Hochberg method.

Supp_Data_16_variance_all.tsv, contains variance scores for all genes during differentiation and regionalization for all modalities Supplementary Fig. 7a

Supp_Data_17_EED_inhib_K27me3_peaks_log2FC.tsv, contains the log2FC of all H3K27me3 peaks upon treatment including the closest gene to each peak (Fig. 5c).

Supp_Data_18_EED_inhib_global_DE.tsv, contains the global differential expression analysis of the DMSO control and EED inhibition. P-values were derived from a likelihood ratio test and were multiple testing corrected using the Benjamini-Hochberg method.

Supp_Data_19_cluster_marker_EED_inhib.tsv, contains all cluster markers of Fig. 5e. P-values were derived from a likelihood ratio test and were multiple testing corrected using the Benjamini-Hochberg method.

Supp_Data_20_EED_inhib_NE_DE.tsv, contains the differential expression analysis of the DMSO control and EED inhibition for the neuroepithelium clusters (Extended Data Fig. 10a). P-values were derived from a likelihood ratio test and were multiple testing corrected using the Benjamini-Hochberg method.

Supp_Data_21_Antibodies, contains an overview of all antibodies used in the study.
