# Supplementary material for: Single-cell epigenomic reconstruction of developmental trajectories from pluripotency in human neural organoid systems
Source: Nat Neurosci. 2024 Jun 24;27(7):1376–86. doi: 10.1038/s41593-024-01652-0 (PMC11239525; doi:10.1038/s41593-024-01652-0)
Supplement: Supplementary file 2 — Reporting Summary [file 41593_2024_1652_MOESM2_ESM.pdf]

nature portfolio

Corresponding author(s): Barbara Treutlein, J. Gray Camp, Fides Zenk

Last updated by author(s): Mar 27, 2024

Reporting Summary

Nature Portfolio wishes to improve the reproducibility of the work that we publish. This form provides structure for consistency and transparency in reporting. For further information on Nature Portfolio policies, see our Editorial Policies and the Editorial Policy Checklist.

Statistics

For all statistical analyses, confirm that the following items are present in the figure legend, table legend, main text, or Methods section.

n/a	Confirmed	
☐	☒	The exact sample size (*n*) for each experimental group/condition, given as a discrete number and unit of measurement
☐	☒	A statement on whether measurements were taken from distinct samples or whether the same sample was measured repeatedly
☐	☒	The statistical test(s) used AND whether they are one- or two-sided<br>*Only common tests should be described solely by name; describe more complex techniques in the Methods section.*
☐	☒	A description of all covariates tested
☐	☒	A description of any assumptions or corrections, such as tests of normality and adjustment for multiple comparisons
☐	☒	A full description of the statistical parameters including central tendency (e.g. means) or other basic estimates (e.g. regression coefficient) AND variation (e.g. standard deviation) or associated estimates of uncertainty (e.g. confidence intervals)
☐	☒	For null hypothesis testing, the test statistic (e.g. *F*, *t*, *r*) with confidence intervals, effect sizes, degrees of freedom and *P* value noted<br>*Give P values as exact values whenever suitable.*
☒	☐	For Bayesian analysis, information on the choice of priors and Markov chain Monte Carlo settings
☒	☐	For hierarchical and complex designs, identification of the appropriate level for tests and full reporting of outcomes
☐	☒	Estimates of effect sizes (e.g. Cohen's *d*, Pearson's *r*), indicating how they were calculated

Our web collection on statistics for biologists contains articles on many of the points above.

Software and code

Policy information about availability of computer code

Data collection: See Methods. No software was used for Data collection.

Data analysis:

Software used in the analysis:
scanpy 1.8.2
kallisto 0.46.0
loompy 3.0.6
scvelo 0.2.4
cellrank 1.3.0
motifmatchr 1.14
cellranger arc 2.0.0
cellranger 5.0.0
CITE-seq-count 1.4.5
seurat 3.2
signac 1.1
tidyverse 1.3.2
Pando 1.0.3

All custom code generated in the study including analysis parameters is available at https://github.com/quadbiolab/organoid_epigenomics.

For manuscripts utilizing custom algorithms or software that are central to the research but not yet described in published literature, software must be made available to editors and reviewers. We strongly encourage code deposition in a community repository (e.g. GitHub). See the Nature Portfolio guidelines for submitting code & software for further information.

Data

Policy information about availability of data

All manuscripts must include a data availability statement. This statement should provide the following information, where applicable:

- Accession codes, unique identifiers, or web links for publicly available datasets
- A description of any restrictions on data availability
- For clinical datasets or third party data, please ensure that the statement adheres to our policy

Raw sequencing data will be deposited to the European Genome Phenome Archive (https://ega-archive.org/). All processed data are available at https://episcape.ethz.ch, where they can be browsed interactively. They can be downloaded at https://doi.org/10.5281/zenodo.10471808.

Research involving human participants, their data, or biological material

Policy information about studies with human participants or human data. See also policy information about sex, gender (identity/presentation), and sexual orientation and race, ethnicity and racism.

Reporting on sex and gender	We have not collected information about sex and gender.
Reporting on race, ethnicity, or other socially relevant groupings	We have not collected or used information about race, ethnicity or other groupings.
Population characteristics	not applicable
Recruitment	Human material was collected fully anonymized and after informed consent.
Ethics oversight	The use of human ES cells for the generation of brain organoids was approved by the ethics committee of northwest and central Switzerland (2019-01016) and the Swiss federal office of public health. Under the Swiss Human Research Act, research performed with fully anonymized human specimens does not require an institutional review for research as long as consent was approved in the first place. The human derived iPCS lines used in the study are available commercially and usage is regulated within the MTA.

Note that full information on the approval of the study protocol must also be provided in the manuscript.

Field-specific reporting

Please select the one below that is the best fit for your research. If you are not sure, read the appropriate sections before making your selection.

☒ Life sciences ☐ Behavioural & social sciences ☐ Ecological, evolutionary & environmental sciences

For a reference copy of the document with all sections, see nature.com/documents/nr-reporting-summary-flat.pdf

Life sciences study design

All studies must disclose on these points even when the disclosure is negative.

Sample size	We used several embryoid bodies/organoids (between 5-100 depending on the size) from 5 different cell lines for each timepoint of the experiment. No statistical methods were used to pre-determine sample sizes, but our sample sizes are similar to those reported in previous publications.
Data exclusions	No datasets were excluded from the study. For single cell analysis we performed strict quality filtering and exclduded individual cells that did contain high quality data. For the details and filtering criteria see: Methods - Data processing for scRNA-Seq and Preprocessing and clustering of scCUT&Tag data
Replication	We used 5 different cell lines and multiple organoids for each timepoint of the timecourse. We demultiplexed them based on SNPs and treated them, therefore, as replicates. For day 60, we processed independent biological replicates, and for day 120, we used the same cell suspension as a technical replicate. All attempts of replication were successful.<br>We validated the enrichment of non-Neuroepithelium cells upon EED-inhibition in two independent organoid batches All bulk Cut&Tag experiments of the inhbitor treated cells were performed in duplicate. All attempts of replication were successful.
Randomization	We randomly chose organoids that we analysed in the timecourse and we randomly selected organoids that were exposed to the inhibitor treatment.
Blinding	Organoids for each timepoint were picked blinded. Full blinding of the experiments on inhibitor treated organoids was not possible due to the phenotypes evident from the organoids development. First computational analysis and inspection of the data were performed blinded.

Reporting for specific materials, systems and methods

We require information from authors about some types of materials, experimental systems and methods used in many studies. Here, indicate whether each material, system or method listed is relevant to your study. If you are not sure if a list item applies to your research, read the appropriate section before selecting a response.

Materials & experimental systems

n/a	Involved in the study
☐	☒ Antibodies
☐	☒ Eukaryotic cell lines
☒	☐ Palaeontology and archaeology
☒	☐ Animals and other organisms
☒	☐ Clinical data
☒	☐ Dual use research of concern
☒	☐ Plants

Methods

n/a	Involved in the study
☒	☐ ChIP-seq
☒	☐ Flow cytometry
☒	☐ MRI-based neuroimaging

Antibodies

Antibodies used

A detailed list of all antibodies used in the study is provided in the materials and methods.
H3K27me3 Diagenode, #C15410195, A0824D
H3K27ac Diagenode, #C15410196, A1723-0041D
H3K27ac GeneTex, GTX50903
H3K4me3 Diagenode, #C15410003, A1052D
H3 Activemotif, #39763, 20418023
β-Catenin BD Bioscience, #610154
guinea pig anti rabbit antibodies online, #ABIN101961
Alexa-Fluor conjugated antibodies ThermoScientific
HRP-conjugated antibodies Jackson ImmunoResearch

Validation

Antibodies used in this study are commercially available and have been validated by the manufacturer. We further validated antibodies against H3K27me3, H3K27ac by Western Blot in the control and upon inhibition of the respective epigenetic writer.
H3K27me3 Diagenode, #C15410195, A0824D (validated by ChIP in E(z)-KD in Zenk et al. 2017, Science and here by EED inhibition Ext. Data Fig. 11b)
H3K27ac Diagenode, #C15410196, A1723-0041D (according to manufacturer validated for ChIP and NGS applications)
H3K27ac GeneTex, GTX50903 (validated by CUT&Tag in CBP-KD embryos in Ciabrelli et al. 2023, Science Advances)
H3K4me3 Diagenode, #C15410003, A1052D (according to manufacturer used in more than 180 publications, validated for ChIP)
H3 Activemotif, #39763, 20418023

Eukaryotic cell lines

Policy information about cell lines and Sex and Gender in Research

Cell line source(s)

A detailed list of all cell lines used in the study is provided in the materials and methods.

Authentication

Cell lines used in the study were authenticated through comparing single nucleotide polymorphisms identified from single cell RNA and CUT&Tag to reference datasets.

Mycoplasma contamination

Regular PCR testing of all cell lines used in the study confirmed that they were free mycoplama contamination.

Commonly misidentified lines
(See ICLAC register)

None.
